# Split diversity in constrained conservation prioritization using integer linear programming

**DOI:** 10.1111/2041-210X.12299

**Published:** 2014-12-06

**Authors:** Olga Chernomor, Bui Quang Minh, Félix Forest, Steffen Klaere, Travis Ingram, Monika Henzinger, Arndt von Haeseler

**Affiliations:** 1 Center for Integrative Bioinformatics Vienna, Max F. Perutz Laboratories, University of Vienna, Medical University ViennaVienna, Austria; 2 Bioinformatics and Computational Biology, Faculty of Computer Science, University of ViennaVienna, Austria; 3 Jodrell Laboratory, Royal Botanic GardensKew, Richmond, UK; 4 Department of Statistics, School of Biological Sciences, University of AucklandAuckland, New Zealand; 5 Department of Zoology, University of OtagoDunedin, New Zealand; 6 Theory and Applications of Algorithms, Faculty of Computer Science, University of ViennaVienna, Austria

**Keywords:** conservation biology, phylogenetic diversity, split diversity

## Abstract

1. Phylogenetic diversity (PD) is a measure of biodiversity based on the evolutionary history of species. Here, we discuss several optimization problems related to the use of PD, and the more general measure split diversity (SD), in conservation prioritization.
2. Depending on the conservation goal and the information available about species, one can construct optimization routines that incorporate various conservation constraints. We demonstrate how this information can be used to select sets of species for conservation action. Specifically, we discuss the use of species' geographic distributions, the choice of candidates under economic pressure, and the use of predator–prey interactions between the species in a community to define viability constraints.
3. Despite such optimization problems falling into the area of NP hard problems, it is possible to solve them in a reasonable amount of time using integer programming. We apply integer linear programming to a variety of models for conservation prioritization that incorporate the SD measure.
4. We exemplarily show the results for two data sets: the Cape region of South Africa and a Caribbean coral reef community. Finally, we provide user-friendly software at http://www.cibiv.at/software/pda.

## Introduction

Many important challenges in biodiversity conservation involve the prioritization of species, habitats or ecosystems for the allocation of limited conservation funding. These problems require techniques that allow the selection of units that maximize a quantity of interest, such as species diversity, phylogenetic diversity or ecosystem function, subject to some number of constraints (e.g. Purvis, Gittleman & Brooks 2005). A basic approach is to focus on taxon richness (Gaston & Spicer 2004) in order to maximize the number of taxa conserved. However, the assumption that all taxa are equally valuable may make taxon richness too simplistic (May 1990).

One approach to incorporating variation among species is to use indices that take into account phylogenetic information (Vanewright, Humphries & Williams 1991; Crozier 1992; Faith 1992), the most popular being phylogenetic diversity (PD; Faith 1992). PD is the amount of evolutionary history encompassed by a given number of taxa (e.g. species) and is often predictive of phenotypic diversity or the ecosystem function provided by a set of taxa (Isaac *et al*. 2007; Cadotte, Dinnage & Tilman 2012; Srivastava *et al*. 2012; Winter, Devictor & Schweiger 2013). Given a phylogenetic tree for a set of taxa, the PD of a taxon subset is calculated as the sum of branch lengths of the minimal subtree spanned by those taxa. PD depends on the availability of a single, reliable phylogenetic tree estimate with branch lengths and cannot readily be calculated when one wishes to use information from multiple trees. The single tree may be a species tree reconstructed from many genes that may have different evolutionary rates (Graur & Li 2000) or even support different tree topologies (Nei 1987). One may instead wish to weigh evidence across these gene trees, or across a number of candidate trees from bootstrap samples (Felsenstein 1985) or from a Bayesian posterior distribution (Yang & Rannala 1997). To resolve this issue, we have recently introduced the concept of split diversity (SD), which generalizes PD by combining information from multiple trees (Minh, Klaere & von Haeseler 2009).

Integer linear programming (ILP; Gomory 1958) is a widely used technique to solve optimization problems in various scientific disciplines (e.g. Jünger *et al*. 2010) with great potential for conservation decision-making. ILP solves problems by optimizing a linear objective function subject to linear constraints acting on integral variables (such as the inclusion or exclusion of species). Theoretically, solving ILP is nondeterministic polynomial-time hard (NP-hard), meaning that to guarantee an optimal solution it may be necessary to evaluate exponentially many subsets of species (e.g. Karp 1972).

With the advances of state-of-the-art ILP software packages such as CPLEX (2012) and GUROBI (2012) (free of charge for academic use) many NP-hard problems can be solved within a reasonable time while ensuring optimal solutions (Jünger *et al*. 2010; and references therein).

Integer linear programming was first applied to the minimum representation problem in biodiversity conservation (Cocks & Baird 1989). Subsequently, ILP has been applied to more complex topics such as minimizing the total land mass protected while maximizing the biodiversity (taxon richness or PD) given limited resources (e.g. Underhill 1994; Possingham, Ball & Andelman 2000; Rodrigues & Gaston 2002; Önal & Briers 2003; Haight & Snyder 2009). Moreover, ILP also works for the more general SD measure (Minh, Klaere & von Haeseler 2010) and predator–prey relationships (Faller 2010). However, to the best of our knowledge, none of the previous work considered species' diet compositions in the ILP formulations.

Here, we further show the efficiency and flexibility of ILP to maximize SD for more generalized conservation questions, the spatial reserve selection and viable taxon selection. We exemplify our approach with two case studies: the Cape of South Africa and the Caribbean coral reef community. We also show that the inclusion of ecological constraints into the ILP formulations is straightforward. Using the GUROBI library, the ILP problems are solved within a few seconds. We provide an implementation in the PDA software package (Minh, Klaere & von Haeseler 2009).

## Materials and methods

### Spatial reserve selection under split diversity

The classical *minimum representation problem* (Cocks & Baird 1989) and *spatial reserve selection* (Moilanen, Wilson & Possingham 2009) assume that one has sufficient resources to protect all taxa (Data S1). However, if the minimal cost required exceeds the allocated budget, one can alternatively minimize the costs such that at least *p*% of the diversity is still preserved. To measure biodiversity, we utilize the concept of split diversity (SD; Minh, Klaere & von Haeseler 2009), but we note that other diversity measures are also applicable.

*Spatial reserve selection* was formulated as integer quadratic programming (Possingham, Ball & Andelman 2000), where one optimizes a quadratic objective function given linear constraints. Further, this problem was discussed and reformulated as ILP (Önal & Briers 2002, 2003). Here, we transform the quadratic objective function into linear function and extend the initial problem to account for SD.

Let *X* = {*s*_*1*_, *s*_*2*_, …, *s*_*n*_} denote the set of *n* taxa of interest. Further, for area *i* ∈ {1, …, *m*} let *R*_*i*_ ⊂ *X* denote the taxa present in area *i*. The question is then

**Problem 1** (*Spatial reserve selection under SD constraints*): Given the taxon set *X*, *m* areas, the costs per unit boundary and a diversity threshold *p*, find the cheapest area subset 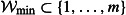 such that the set of taxa preserved in *W*_min_ must constitute at least *p*% of the total SD of *X*.

We now formulate Problem 1 in ILP parlance. To this end, we construct the *m × n* presence/absence matrix 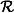, where *r*_*ij*_ = 1 if taxon *s*_*j*_ is present in area *i*, and *r*_*ij*_ = 0 otherwise. Moreover, we encode an arbitrary subset 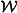 of areas by the binary vector (*x*_1_, *x*_2_,…, *x*_*m*_), where *x*_*i*_ = 1 if 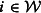, and *x*_*i*_ = 0 otherwise.

The *spatial reserve selection* includes the area conservation costs (e.g. acquisition and maintenance costs of the area) and boundary costs in terms of the cost of fencing the entire selected areas, where high boundary costs prevent highly fragmented areas. For area *i* ∈ {1, …, *m*}, we denote its cost by *c*_*i*_. To quantify the boundary length, we define a symmetric *m* × *m* matrix with each entry *b*_*ij*_ being the shared boundary length between area *i* and *j*. If *i* and *j* are not adjacent we set *b*_*ij*_ = 0. Finally, *b*_*ii*_ equals the perimeter of *i*. β defines the conservation cost per unit boundary length.

For each pair of adjacent areas *i* and *j* (*b*_*ij*_ > 0), we introduce variable *z*_*ij*_, where *z*_*ij*_ = 1 if *x*_*i*_ = *x*_*j*_ = 1 and *z*_*ij*_ = 0, otherwise (i.e., 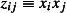).

The linear function equivalent to quadratic objective function in Possingham, Ball & Andelman (2000) is given by

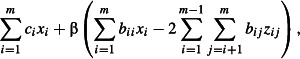

where variables *z*_*ij*_ are constrained by *z*_*ij*_ ≤ *x*_*i*_ and 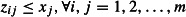, which ensures that *z*_*ij*_ = *x*_*i*_*x*_*j*_ Note that the number of introduced variables *z*_*ij*_ is typically much smaller than 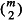 as only pairs of spatially adjacent areas are relevant.

To introduce SD constraints, we follow the notations of Minh, Klaere and von Haeseler (2010) by denoting an input split system as (Σ, λ) where Σ is a set of splits (bipartitions of *X*) and λ the split-weight function. A split σ* *∈* *Σ is represented by an *n*-element binary vector (σ_1_, σ_2_,…, σ_n_), where *n* is the number of taxa and σ_*i*_ takes a value of 0 or 1 depending on the bipartition that *s*_*i*_ belongs to. Note that the vector (1 − σ_1_, 1 − σ_2_,…, 1 − σ_n_) represents the same split.

For an area subset 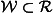, denote *X|w* the subset of taxa present in at least one area in *W*. To compute SD of *X|w* based on the split system (Σ, λ), we introduce a so-called split variable *y*_σ_ for every split σ, where *y*_σ_ = 1 if σ separates at least two taxa of *X|w*, and *y*_σ_ = 0 otherwise. It then follows that

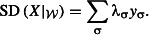


Finally, problem 1 is formulated as:

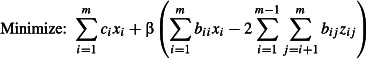
eqn 1.1

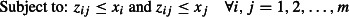
eqn 1.2

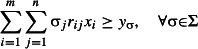
eqn 1.3

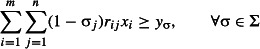
eqn 1.4

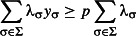
eqn 1.5

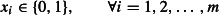


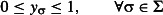


The objective function (1.1) together with constraints (1.2) is equivalent to that of the spatial reserve selection. The so-called split constraints (1.3 and 1.4) determine the values of *y*_σ_. The inequality (1.5) ensures the conservation target *p*% of SD.

### Taxon selection under viability constraints

We now turn to another problem that arises when predator–prey interactions between species are incorporated into conservation decisions. If the candidate species for prioritization depend on each other, as in a food web representing the predator–prey relationships among community members, our prioritization can account for such information. For example, we may wish to select a set of taxa *S* with maximal diversity under the constraint that these taxa form a *viable* food web (Moulton, Semple & Steel 2007). Here, we focus on the bottom-up dependencies represented in food webs, so that a taxon is defined as viable in *S* if it is either a basal taxon in the food web (i.e. a species without prey such as a primary producer) or a predator that has at least one prey in *S. S* is called viable if all its taxa are viable. The problem is now formulated as:

**Problem 2** *(Viable taxon selection)*: Given a food web and a phylogenetic tree, choose a viable subset of at most *k* taxa, which maximizes PD.

Problem 2 has been solved using ILP (Faller 2010). In the following, we will further generalize the problem from PD to SD (Problem 3) and propose an extended definition of viability that includes diet composition (Problem 4).

### Extension to SD

**Problem 3** *(Viable taxon selection under SD)*: Given a food web and a split system (Σ, λ), choose a viable subset of at most *k* taxa, which maximizes SD.

We transform Problem 3 into an ILP. To this end, we introduce for each taxon *s*_*i*_ ∈ *X* a taxon variable *v*_*i*_. A subset *S* ⊂ *X* is represented by a vector (*v*_1_,…, *v*_*n*_), where *v*_*i*_ = 1 if *s*_*i*_ ∈ S and *v*_*i*_ = 0 if *s*_*i*_ ∉ *S*. For each split σ ∈ Σ, we introduce a split variable *y*_σ_, where *y*_σ_ = 1 if σ separates at least two taxa in *S*, and *y*_σ_ = 0 otherwise.

Following the notation of Moulton, Semple & Steel (2007), we denote by D = (*X, A*) a directed acyclic graph representing the food web, where *A* denotes the set of arrows (directed edges) represented as a pair of taxa, s.t. (*s*_*i*_*, s*_*j*_) ∈ *A* if taxon *s*_*i*_ feeds on *s*_*j*_. We denote by *C*_*i*_ the set of preys of *s*_*i*_. If *C*_*i*_ = ∅, *s*_*i*_ is called a *basal prey*.

Problem 3 is then equivalent to:

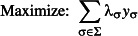
eqn 3.1

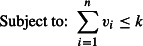
eqn 3.2

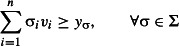
eqn 3.3

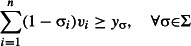
eqn 3.4

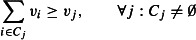
eqn 3.5

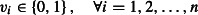


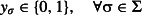


The resulting solution (*v*_1_, …, *v*_*n*_) corresponds to a set *S*_max_, which is ensured by constraint (3.2) to contain at most *k* taxa. Preservation of split σ_*i*_ is provided by the constraints (3.3 and 3.4) and viability of a subset is assured by constraint (3.5).

If we want some of the taxa to be included in *S*_max_ irrespective of constraints, we simply set the corresponding *ν*_*j*_ = 1.

### Extension to account for diet composition

Problems 2 and 3 consider a predator as a viable member of a food web even if only one of its prey taxa is conserved. However, if the conserved prey taxon makes up only a small fraction of the predator's diet, the predator is unlikely to maintain sufficient food intake to be treated as a viable species. For that reason, we introduce a more realistic definition of viability that considers the diet composition of predators. To this end, we denote by *D* = (*X, W*) a weighted food web of the taxon set *X*, where *W* is the diet composition matrix. Here, we weight the arrow (*s*_*j*_, *s*_*i*_) of the food web by *w*_*ij*_, the proportion of prey *s*_*i*_ in the diet of predator *s*_*j*_, such that the diet composition for each predator sums up to 100%, (i.e. 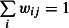 for every predator *s*_*j*_).

Using (*X*, *W*), we compute the total diet of a predator *s*_*j*_ over all of its prey taxa in a set *S* as:

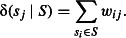


This allows us to set a constraint that each predator must have a minimum proportion of its prey composition preserved for a set of taxa to be viable. We define a subset *S* of taxa as *d%-viable* if every predator *s*_*j*_ ∈ *S* has the score δ (*s*_*j*_|*S*) ≥* d*.

**Problem 4** *(d%-viable taxon selection under SD)*: Given a weighted food web *D* = (*X*, *W*) and a split system (Σ, λ), select a *d%–*viable subset of at most *k* taxa, which maximizes SD.

Problem 4 is again solved with ILP by simply modifying constraint (3.5) in Problem 3 to:

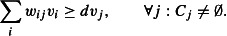


### Software availability

We provide a user-friendly software package PDA (Minh, Klaere & von Haeseler 2009), freely available at http://www.cibiv.at/software/pda, to carry out conservation prioritization analysis based on PD and SD including various constraints. The user inputs a phylogenetic tree or a split network and additional information relevant for the conservation decision problem (i.e. areas data, costs for areas, costs for species, weighted or non-weighted dependency networks such as food webs). As a result, PDA outputs optimal taxon or area sets selected by ILP and detailed information about the sets. Additional details are explained in the user manual.

## Results

### Case study I: The cape of South Africa

We analyse a data set consisting of 735 flowering plant genera (Forest *et al*. 2007; Data S1) distributed over 201 quarter-degree squares (QDS; ca. 25 × 27 km^2^) of the Cape of South Africa, a biodiversity hotspot (Myers *et al*. 2000). The Cape region is a small area (ca. 90 000 km^2^) in the southernmost part of the African continent and is one of the most botanically species-rich areas of the world with more than 9000 species, of which almost 70% are endemic (Goldblatt & Manning 2002). This especially rich biodiversity has been extensively documented (Goldblatt & Manning 2002; Linder 2003, 2005) and its importance as one of the major repositories of global biological diversity has been widely acknowledged (Linder 2001; Kuper *et al*. 2004; Mittermeier *et al*. 2005; Kreft & Jetz 2007). Among the 735 genera in this data set, 274 include at least one species classified as vulnerable, endangered or critically endangered (Raimondo *et al*. 2009); these will be referred here as threatened genera. Of the 274 threatened genera, 17 belong to the top-20 most-threatened genera of South Africa, based on the proportion of their species that are threatened (Raimondo *et al*. 2009).

### QDS selection under split diversity

The minimum representation and spatial reserve selection (for the analysis, see Data S3) are solely based on the concept of genus richness. However, it was shown that genus richness and PD are decoupled in the Cape of South Africa and that PD is more appropriate than genus richness in certain conservation scenarios (Forest *et al*. 2007). Here, we take one step further by considering split diversity (Minh, Klaere & von Haeseler 2009) across the 100 bootstrap trees based on the *rbcL* sequence alignment (Data S2). Moreover, we extend the reserve selection to account for a diversity constraint that at least *p*% of SD must be preserved, which can be solved again with ILP (Problem 1; Materials and Methods). If *p* = 100%, the problem is identical to the spatial reserve selection. If *p* < 100%, we only protect a fraction of taxa, needing only a fraction of the areas. Such a scenario is typically applied when the available budget does not suffice to save 100% of diversity.

**Fig 1:**
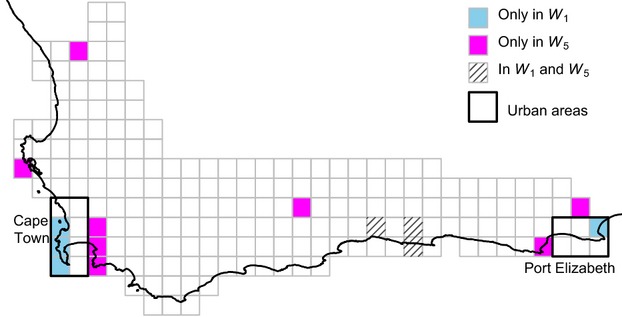
QDS selected to conserve 95% of split diversity under uniform conservation cost (*W*_1_) and under prioritization of rural areas (*W*_5_).

To conserve *p* = 95%, only seven QDS are necessary (depicted in blue and hatched; 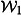 in Fig. 1), while for *p* = 100% 28 QDS were selected (Fig. S1). The selected QDS cover the Cape Peninsula (three QDS), part of the afrotemperate forests along the southern coast (three QDS) and one QDS in the Port Elizabeth area. Notably, the total cost in terms of area, which shall be incurred when 95% of the SD is conserved, is 2410 km^2^, less than one-fifth of the area needed for a conservation goal of 100% (Fig. S1). This means saving the last 5% of diversity needs four times the budget required by the first 95%.

### Impact of economic pressure

**Fig 2:**
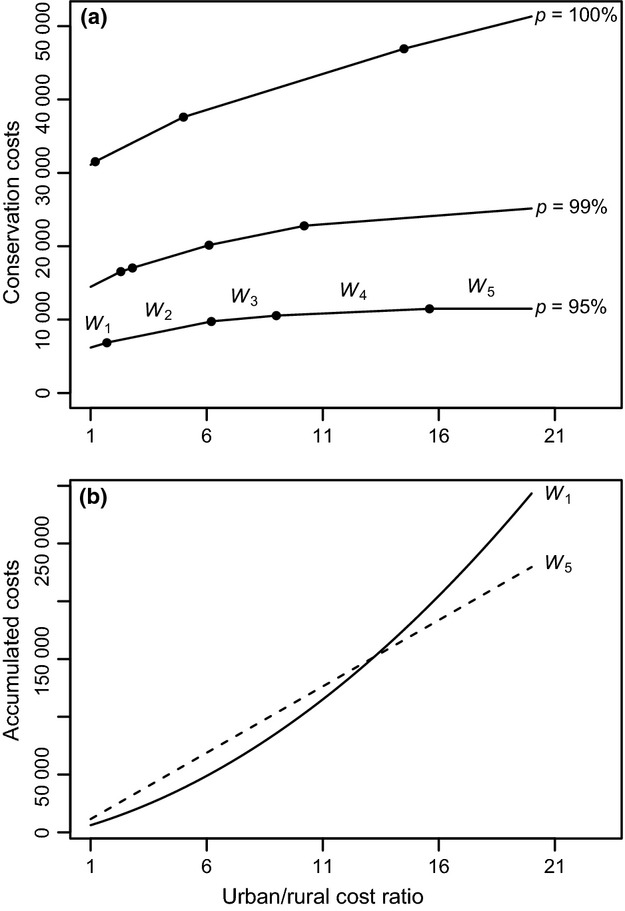
(a) Minimal costs to conserve *p* = 95%, 99% and 100% of split diversity with varying urban/rural cost ratios. The points on the curves indicate the change in the optimal sets of QDS found by ILP. For *p *=* *95%, we identified five optimal sets denoted by *W*_1_ to *W*_5_. Also note that preserving the last 1% of diversity more than doubles the conservation cost. (b) Accumulated costs for optimal sets of QDS *W*_1_ and *W*_5_ as the cost ratios gradually increase over time.

The reserve selections presented so far implicitly assume uniform conservation cost per km^2^. This is unrealistic since it is more expensive to establish reserves in the vicinity of big cities (Cape Town and Port Elizabeth) than in rural areas. We therefore extend the model by increasing the conservation cost per km^2^ of QDS in the urban areas relative to those of rural areas. The cost ratio between the urban and rural areas represents the economic pressure put on urban areas. Because the ILP solution can be rapidly computed, we could analyse several different scenarios. As QDS close to Cape Town and Port Elizabeth are defined here as more expensive, the conservation cost increases for fixed *p* (Fig. 2a).

Because we want to conserve a certain fraction of current diversity at minimal costs, sometimes the selected QDS-set will change. Each interval of the curves in Fig. 2a reflects a selected set of QDS. For example, for *p* = 95%, five different sets of optimal QDS are identified and denoted as *W*_1_ to *W*_5_ (Fig. 2a and Table 1). The selected QDS in *W*_1_ to *W*_5_ gradually move away from the cities (Table 1 and Fig. S3). For price ratios larger than 15·6, all selected QDS (*W*_5_) are outside urban regions and a further increase of costs next to big cities does not affect the conservation decision, indicated by a slope of zero (Fig. 2a). Minimizing costs has different effects on the conservation success of threatened genera (Table 1). If we consider the most-threatened genera, then irrespective of the costs we will always lose *Polhillia* (Leguminosae). This genus occurs in 8 QDS (Fig. S4), which are never included in the selected QDS. Nevertheless, we always preserve 585 (ca. 80%) genera regardless of the cost ratios, 235 (ca. 86%) of which are threatened.

**Table 1:** Features of the five optimal sets of QDS (*W*_1_ to *W*_5_) to preserve 95% of split diversity under different urban/rural cost ratios. The sets of QDS for *W*_2_, *W*_3,_ *W*_4_ can be found in Fig. S3

Urban/rural cost ratio range	1–1·7	1·8–6·2	6·3–9·0	9·1–15·6	>15·6
QDS-set	*W*_1_ (Fig. 1)	*W*_2_	*W*_3_	*W*_4_	*W*_5_ (Fig. 1)
#QDS	7	7	9	9	11
#Urban QDS	4	3	3	1	0
Area (km^2^)	2410	2150	3200	3805	4970
#Genera	648	656	660	657	662
#Threatened genera	244	249	254	253	250
Most-threatened genera that are not conserved	*Polhillia* *Clivia* *Daubenya* *Marasmodes*	*Polhillia* *Clivia* *Daubenya*	*Polhillia*	*Polhillia* *Clivia* *Daubenya*	*Polhillia* *Clivia* *Daubenya*

Figure 2a allows a second interpretation of the costs for biodiversity efforts that will lead to economically more sustainable conservation decisions. If we take the urban/rural cost ratio as the economic pressure put on the urban areas in the future, then we can read the graph as the extrapolation of running costs incurred in the future for a fixed value of *p*. For each set of QDS selected the costs to protect will increase linearly with increasing price ratio (this follows from the ILP formulation of the problem). The total costs that will accumulate over the years will therefore grow quadratically. The only exceptions are selected areas where the conservation costs are independent of the price ratio, like *W*_5_ for *p* = 95%. However, the conservation costs at present are 11 482 virtual price units for *W*_5_ compared with 6186 virtual price units for *W*_1_. But in the long run, the accumulated costs will be lower for *W*_5_ (Fig. 2b). Therefore, given the prediction about the economic pressure imposed on urban areas, it may be better to select *W*_5_ right from the beginning, because the most inexpensive selection (*W*_1_) will already accumulate more costs if the price ratio is larger than 13·2 (Fig. 2b).

### Case study II: Caribbean coral reef community

The second case study demonstrates how predator–prey interactions can be incorporated in the analysis used for conservation prioritization. We examine a food web representing the predator–prey relationships of 242 taxa (mostly species) and 6 aggregated trophic groups from a Caribbean coral reef community (Table S1).

Of the 248 nodes in the food web, all but the four basal nodes depend on consumption of at least one other taxon, and all but one (tiger shark, *Galeocerdo cuvier*) is prey for at least one other taxon. The food web is characterized by a complex structure and extensive omnivory, with food chains of as many as 25 links. Thus, this ecological network features extensive and complex dependencies among species that must be accounted for if we are to select a viable subset of taxa.

For the 242 taxa, we obtained sequences for six distinct genes if available (Table S3), computed for each gene a multiple sequence alignment and reconstructed six maximum likelihood (ML) trees (Fig. S7), that served as input to infer a split system (Σ, λ) (Data S2 and Fig. S9). We also computed the ML tree *T* (Fig. S8) from the concatenated alignments. We used *T* and (Σ, λ) to compute PD and SD, respectively. The split system (Σ, λ) contains 558 non-trivial splits (i.e. splits that contain at least two taxa on either side), which is 2·3 times more splits than *T*. This indicates that the six gene trees are incongruent. This incongruence has a number of potential causes, including insufficient phylogenetic information, noise in the alignments or even non-treelike evolution (Doolittle 1999; Philippe *et al*. 2011).

We now discuss the optimal taxon sets obtained under different constraints. We require that the aggregate trophic groups are always included in the optimal sets, because they are at the base of the food web and because they represent taxonomically diverse collections of organisms rather than defined taxa.

Maximizing PD or SD without taking into account the food web leads to inviable sets, where *Synodus foetens* and *Antennarius striatus* do not find prey (Data S3 and Fig. S5). Therefore, in the following, we require that the optimal SD set is viable (i.e. each predator must have at least one prey in the set). The problem is then called Viable Taxon Selection under SD (Problem 3).

**Fig 3:**
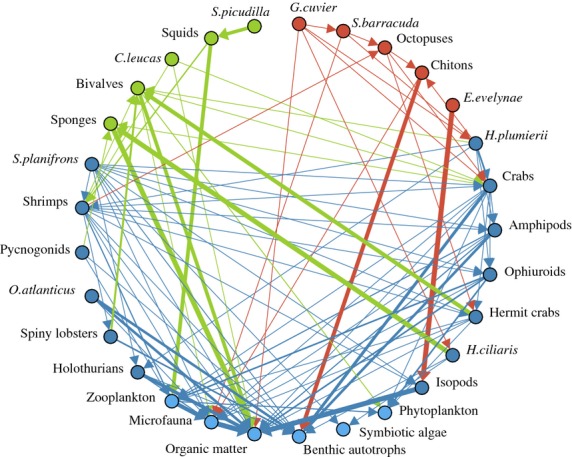
Food web restricted to only those taxa present in *S*_1_ or *S*_2_ (see main text). Red, green and blue nodes depict the taxa present exclusively in *S*_1_, exclusively in *S*_2_, and in both sets, respectively. Light blue nodes correspond to aggregated groups. Arrows connect from predators to their preys with thickness reflecting the prey proportion in the predator diet. Arrows pointing to or from green and red nodes are coloured green and red respectively. Arrows between blue nodes are coloured blue. Note that the arrows between green and red nodes are ignored.

The resulting optimal set denoted *S*_1_ (red and blue nodes; Fig. 3) containing 10% of the taxa has a relative SD of 57·67% compared with the total SD of all taxa (i.e. only 0·22% less than the taxon-set chosen solely on SD). This loss in relative diversity is due to the replacement of *Antennarius striatus* (the ‘non-viable’ species, Data S3) with *Elacatinus evelynae*. Therefore, *S*_1_ ‘repairs’ the viability with negligible loss of diversity.

We now look in more detail at the diet composition of the species in *S*_1_. For each predator *s*_*j* _∈ *S*_1_, we compute its proportion of diet conserved in 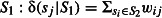, where 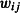 is the proportion of prey *s*_*i*_ in the diet of *s*_*j*_ (Materials and Methods). For the taxa *Galeocerdo cuvier*, octopuses*, Sphyraena barracuda* and *Holacanthus ciliaris* (four red nodes; Fig. 3) only 11%, 7%, 6% and 2% of their diet is conserved, while for pycnogonids*,* hermit crabs*,* spiny lobsters and *Haemulon plumierii* the diet proportion conserved ranges between 20% and 50% (Table S4).

The above observations indicate that the simple viability constraint (conserving at least one prey per predator) might result in some predators having an insufficient availability and variety of prey. To address this, we applied the *d*%-viability constraint, which requires that every taxon in the optimal SD set must have at least *d*% of its diet composition conserved (Materials and Methods). Note that the *d*%-viability constraint reduces to the simple viability constraint if we set *d* = ∈ (i.e. a very small number). Despite this additional constraint, the problem of *d*%-viable taxon selection can still be solved by ILP (Problem 4, Materials and Methods).

As an illustration, for *k *=* *24 and *d *=* *30%, the optimal set *S*_2_ (green and blue nodes; Fig. 3) has a relative SD of 56·36%, a reduction of 1·31% compared with *S*_1_. Moreover, more stringent viability constraints with higher *d* still provide almost equally optimal subsets (Data S3 and Fig. S6).

*S*_2_ has five species (green nodes; Fig. 3) not present in *S*_1_. At the same time, five taxa (red nodes; Fig. 3) are not present in *S*_2_, of which *G. cuvier,* octopuses and *S. barracuda* have less than 30% diet conserved by their preys in *S*_1_. On the other hand, pycnogonids*, H. ciliaris* and hermit crabs, which have less than 30% diet conserved in *S*_1_, now become 30%-viable in *S*_2_. This is because the newly added taxon (bivalves; green node; Fig. 3) is a prey of hermit crabs, contributing 80% to their diet. Another new taxon (sponges) being a prey of pycnogonids and *H. ciliaris* contributes to their diets 15% and 97% respectively, making them 30%-viable.

### Computational time and optimality

The computational time to solve all the problems with the PDA software was less than 8 s on a 2·66 GHz computer. 98% of the runs for different parameters of Problem 4 consumed less than 1 s with a maximum of 3 s. Simulations with varying complexities of split networks and food webs yielded average run times of 2 s, with a maximum of 8 s.

We also applied the software Marxan 2.1.1 (Ball, Possingham & Watts 2009; Data S3) to the minimum representation and spatial reserve selection (subproblems of Problem 1) for the first case study. Marxan found optimal solutions in only 7/50 runs and 3/50 runs, respectively. Marxan also required 20 min for 50 runs. Therefore, Marxan does not guarantee optimal solutions while requiring more computations than PDA.

## Discussion

We have presented ILP solutions for various biodiversity optimization problems that incorporate SD and include economic and ecological constraints. The first advantage of ILP compared to other approaches is its flexibility: we can account for a variety of constraints while using the same basic formulations. While many other computational techniques have been introduced for some of the problems presented (Chernomor *et al*. 2014; and references therein), here, we further show that ILP provides a general framework to address many more problems without much extra effort. Therefore, the PDA software provided here complements existing conservation prioritization tools such as Marxan or Zonation (Moilanen, Kujala & Leathwick 2009).

The second advantage of ILP is the computational efficiency and the guarantee of optimality. Thanks to the powerful GUROBI solver employed, the analyses were carried out within seconds (Data S3). This is contrary to the computing times reported by Pressey, Possingham & Margules (1996) but in accordance with others (Rodrigues & Gaston 2002; Önal 2004). The different time requirements might be explained by inefficient ILP solvers (LP_SOLVE and LINGO) whereas Rodrigues & Gaston (2002) used the well-known CPLEX library (see also Önal 2004).

We demonstrated the practical utility of ILP with two large-scale case studies: the flora of the Cape region of South Africa and the Caribbean reef food web. In the first case study, the inclusion of the increasing protection costs in urban areas acts as a paradigm of how future developments may influence present day decisions. Such a model suggests a long-term conservation goal that appears expensive for the time being but will be more sustainable in the future. Admittedly, one limitation of the Cape region example is the simple cost model, mainly due to the unavailability of land price data. However, more complex cost indices can be easily incorporated thanks to the flexibility of the ILP paradigm. Further limitations include the somewhat coarse nature of the data (i.e. genus-level phylogenetic trees and quarter-degree square distribution data). The latter may be inappropriate for the conservation purposes due to the large area covered by a QDS (ca 675 km^2^) and the heterogeneity of the landscape in the Cape of South Africa. This situation leads to a relatively high species turnover along environmental and geographical gradients (Cowling, Holmes & Rebelo 1992).

The Caribbean food web example demonstrates the usage of viability constraints in conservation prioritization thanks to the availability of food web and diet composition data. Such food webs allow us to analyse an entire set of species as an interaction network rather than as isolated units. We find that in the case of the Caribbean food web, including viability constraints results in only small reductions in the amount of biodiversity that can be preserved. This is explained by the fact that the most evolutionarily distant taxa are concentrated on the low trophic levels of the food web. Therefore, by maximizing PD or SD for the Caribbean community, we already obtained almost viable sets. However, taxon selection based on viability also highlighted which representatives of each subclade contribute to viability of the set. In practice, incorporating viability constraints has the potential to prevent the use of limited resources on specialist taxa unless a sufficient resource base to support them is also preserved.

While the incorporation of predator–prey links and diet composition gets us closer to ecological realism, there are nonetheless many factors that are not accounted for in the examples described here. First, we are only considering predator–prey relationships, and not other interaction types such as mutualism, facilitation or interference competition (Kefi *et al*. 2012). This framework should be applicable to other types of interaction networks, such as mutualism networks, that allow viability criteria to be specified. For example, a viable taxon may require the preservation of at least one mutualist partner sufficient to contribute a certain fraction of mutualist benefit. We also consider only the bottom-up dependencies within food webs, not top-down effects of predators on their prey (e.g. apparent competition, trophic cascades). The proper incorporation of the complexity of interactions that result from top-down effects may require a move from a static representation of a food web to a population-dynamic model that explicitly includes extinction due to population decline (Ebenman, Law & Borrvall 2004). However, this is beyond the scope of this paper.

One may also need to consider how and if it is appropriate to incorporate diet composition to ensure that each taxon has at least *d*% of its food base preserved. Most published food webs contain only a topological representation of predator–prey relationships, and large food webs such as the Caribbean data set that include weights representing energy flow or diet composition are rare. However, even in the absence of diet composition data, one has the option of assigning the links between a predator and each of its *n* prey a weight of 1/*n*, assuming that they are of equal importance. This allows the application of additional criteria; for example, that a viable predator must have access to at least 50% of its prey taxa. Where diet composition data are available, they provide a means of indirectly considering taxon abundances, as more abundant taxa will generally make up a greater proportion of their predators’ diets. Further, if some prey types are only available during certain seasons, one could devise ‘seasonal constraints’ (similar to area constraints) ensuring that some prey taxa are present for every season. Finally, one could consider contributions from preys that do not appear in the food web (e.g. preys consumed outside the spatial area covered by the food web data) by crediting these predators with some proportion of their prey intake regardless of the taxon set selected. Such additional constraints can be easily included in the ILP framework.

The set of taxa returned by the optimization procedure is a starting point for conservation planning, but should be followed by consideration of the biology of the selected taxa. A food web is a simplified representation of a community or meta-community and lacks information that might bear on the suitability of the taxon set. For example, it should be confirmed that the prey taxa predicted to support each predator are sufficiently abundant and widespread to do so, or that they can reasonably be expected to become more common as a result of conservation action. If the food web contains errors, such as a link between taxa that no longer co-occur or the omission of an important link, it might lead to suboptimal taxon selection. Further, taxa may be subject to additional constraints that may be difficult to capture in the ILP, so at times it may be necessary to reconsider the taxa targeted for conservation action in light of additional biological or societal information.

We note that the ILP framework is extensible to other diversity measures provided that the measures can be expressed as a linear function, for example the number of segregating sites (Watterson 1975; Bryant & Klaere 2012). Moreover, the spatial reserve selection under SD (Problem 1) can be extended to take into account abundance data (e.g. the population size of plant species). The constraint is then to preserve at least the minimum abundance required for the persistence of each taxon. Another extension to the viable taxon selection problems 2, 3 and 4 is to choose species under budgetary constraints. Here, each species has a conservation cost and the inclusion of the taxon is constraint by the budget. Close collaboration between conservation biologists and mathematicians is recommended to convert complex conservation problems into an ILP framework (see also Underhill 1994).

The importance of preserving the diversity of life is widely recognized and understood. In an ideal world, we could ensure the persistence of all levels of biodiversity, but with limited resources the prioritization of some taxa or ecosystems is unavoidable. We thus need good criteria with which to apply triage, to prioritize the allocation of these resources to maximize conservation return under budget constraints (Bottrill *et al*. 2008). We have demonstrated the utility of the ILP approach to show how sensible and objective conservation decisions can be made in a world of limited resources, numerous economical and ecological constraints. The evaluation of different future scenarios with the aid of the ILP approach presented here will certainly prove to be a valuable contribution to conservation planning in a changing world.

## Data accessibility

All data used in this manuscript are present in the manuscript and its supporting information.

## Supporting Information

Data S1 Reserve Selection Problems Revisited. **Data S2.** Data description. **Data S3.** Additional results and analysis. **Table S1.** Diet composition matrix for 250 Caribbean species. **Table S2.** NCBI accession numbers of six genes for Caribbean case study. **Table S3.** Sequence data collected for Caribbean case study. **Table S4.** List of taxa in optimal sets *S*_*PD*_, *S*_*SD*_, *S*_1_, *S*_2_ for Caribbean case study. **Fig. S1.** QDS chosen by the minimum representation problem and the spatial reserve selection for the Cape of South Africa. **Fig. S2.** QDS determined by Marxan for the minimum representation problem. **Fig. S3.** QDS for *W*_2_, *W*_3_, *W*_4_. **Fig. S4.** QDS that host the threatened genera *Polhillia*, *Clivia*, *Daubenya*, and *Marasmodes*. **Fig. S5.** Food web restricted to only those taxa present in *S*_*PD*_ or *S*_*SD*_. **Fig. S6.** Dependence of SD on subset size, *k*, and diet portion, *d*. **Fig. S7.** Gene trees for 12S rRNA, 16S rRNA, 18S rRNA, 28S rRNA, COI, CYTB. **Fig. S8.** Tree for supermatrix of six genes. **Fig. S9.** Super split network of six gene trees.

## References

[b1] Ball IR, Possingham HP, Possingham HP, Watts M, Moilanen A, Wilson KA (2009). Marxan and relatives: Software for spatial conservation prioritisation. Spatial Conservation Prioritisation: Quantitative Methods and Computational Tools.

[b2] Bottrill MC, Joseph LN, Carwardine J, Bode M, Cook C, Game ET (2008). Is conservation triage just smart decision making?. Trends in Ecology & Evolution.

[b3] Bryant D, Klaere S (2012). The link between segregation and phylogenetic diversity. Journal of Mathematical Biology.

[b4] Cadotte MW, Dinnage R, Tilman D (2012). Phylogenetic diversity promotes ecosystem stability. Ecology.

[b5] Chernomor O, Klaere S, von Haeseler A, Grandcolas P, Minh BQ, Pellens R (2014). Split diversity: measuring and optimizing biodiversity using phylogenetic split networks. Biodiversity Conservation and Phylogenetic Systematics.

[b6] Cocks KD, Baird IA (1989). Using mathematical-programming to address the multiple reserve selection problem – An example from the Eyre Peninsula, South-Australia. Biological Conservation.

[b7] Cowling RM, Holmes PM, Cowling RM, Rebelo AG (1992). Ecology of Fynbos: Nutrients, Fire and Diversity.

[b8] CPLEX (2012).

[b9] Crozier RH (1992). Genetic diversity and the agony of choice. Biological Conservation.

[b10] Doolittle WF (1999). Phylogenetic classification and the universal tree. Science.

[b11] Ebenman B, Law R, Borrvall C (2004). Community viability analysis: the response of ecological communities to species loss. Ecology.

[b12] Faith DP (1992). Conservation evaluation and phylogenetic diversity. Biological Conservation.

[b13] Faller B (2010).

[b14] Felsenstein J (1985). Confidence-limits on phylogenies – an approach using the bootstrap. Evolution.

[b15] Forest F, Grenyer R, Rouget M, Davies TJ, Cowling RM, Faith DP (2007). Preserving the evolutionary potential of floras in biodiversity hotspots. Nature.

[b16] Gaston KJ, Spicer JI (2004). Biodiversity: An Introduction.

[b17] Goldblatt P, Manning JC (2002). Plant diversity of the Cape region of Southern Africa. Annals of the Missouri Botanical Garden.

[b18] Gomory RE (1958). Outline of an algorithm for integer solutions to linear programs. Bulletin of the American Mathematical Society.

[b19] Graur D, Li W-H (2000). Fundamentals of Molecular Evolution.

[b20] GUROBI (2012).

[b21] Haight RG, Possingham HP, Snyder SA, Moilanen A, Wilson KA (2009). Integer programming methods for reserve selection and design. Spatial Conservation Prioritization: Quantitative Methods and Computational Tools.

[b22] Isaac NJB, Turvey ST, Collen B, Waterman C, Baillie JEM (2007). Mammals on the EDGE: conservation priorities based on threat and phylogeny. PLoS ONE.

[b23] Jünger M, Liebling TM, Naddef D, Nemhauser GL, Pulleyblank WR, Reinelt G, Rinaldi G, Wolsey LA (2010). 50 Years of Integer Programming 1958–2008: From the Early Years to the State-of-the-Art.

[b24] Karp R, Bohlinger J, Miller R, Thatcher J (1972). Reducibility among Combinatorial Problems. Complexity of Computer Computations.

[b25] Kefi S, Berlow EL, Wieters EA, Navarrete SA, Petchey OL, Wood SA (2012). More than a meal… integrating non-feeding interactions into food webs. Ecology Letters.

[b26] Kreft H, Jetz W (2007). Global patterns and determinants of vascular plant diversity. Proceedings of the National Academy of Sciences of the United States of America.

[b27] Kuper W, Sommer JH, Lovett JC, Mutke J, Linder HP, Beentje HJ (2004). Africa's hotspots of biodiversity redefined. Annals of the Missouri Botanical Garden.

[b28] Linder HP (2001). Plant diversity and endemism in sub-Saharan tropical Africa. Journal of Biogeography.

[b29] Linder HP (2003). The radiation of the Cape flora, Southern Africa. Biological Reviews of the Cambridge Philosophical Society.

[b30] Linder HP (2005). Evolution of diversity: the Cape flora. Trends in Plant Science.

[b31] May RM (1990). Taxonomy as destiny. Nature.

[b32] Minh BQ, Klaere S, von Haeseler A (2009). Taxon selection under split diversity. Systematic Biology.

[b33] Minh BQ, Klaere S, Hirota K, von Haeseler A, Pham SB, Hoang TH, McKay B (2010). SDA*: A simple and unifying solution to recent bioinformatic challenges for conservation genetics. The Second International Conference on Knowledge and Systems Engineering.

[b34] Mittermeier RA, Gil PR, Hoffman M, Pilgrim J, Brooks T, Mittermeier CG, Lamoreux J, Fonseca GABD (2005). Hotspots Revisited: Earth's Biologically Richest and Most Endangered Terrestrial Ecoregions.

[b35] Moilanen A, Kujala H, Possingham HP, Leathwick JR, Moilanen A, Wilson KA (2009). The Zonation framework and software for conservation prioritization. Spatial Conservation Prioritization: Quantitative Methods and Computational Tools.

[b36] Moilanen A, Wilson KA, Possingham HP (2009). Spatial Conservation Prioritization: Quantitative Methods and Computational Tools.

[b37] Moulton V, Semple C, Steel M (2007). Optimizing phylogenetic diversity under constraints. Journal of Theoretical Biology.

[b38] Myers N, Mittermeier RA, Mittermeier CG, da Fonseca GAB, Kent J (2000). Biodiversity hotspots for conservation priorities. Nature.

[b39] Nei M (1987). Molecular Evolutionary Genetics.

[b40] Önal H (2004). First-best, second-best, and heuristic solutions in conservation reserve site selection. Biological Conservation.

[b41] Önal H, Briers RA (2002). Incorporating spatial criteria in optimum reserve network selection. Proceedings of the Royal Society B-Biological Sciences.

[b42] Önal H, Briers RA (2003). Selection of a minimum-boundary reserve network using integer programming. Proceedings of the Royal Society B-Biological Sciences.

[b43] Philippe H, Brinkmann H, Lavrov DV, Littlewood DTJ, Manuel M, Worheide G, Baurain D (2011). Resolving difficult phylogenetic questions: why more sequences are not enough. Plos Biology.

[b44] Possingham HP, Ball IR, Burgman M, Andelman S, Ferson S (2000). Mathematical methods for identifying representative reserve networks. Quantitative Methods for Conservation Biology.

[b45] Pressey RL, Possingham HP, Margules CR (1996). Optimality in reserve selection algorithms: when does it matter and how much?. Biological Conservation.

[b46] Purvis A, Gittleman JL, Brooks T (2005). Phylogeny and Conservation.

[b47] Raimondo D, Staden LV, Foden W, Victor JE, Helme NA, Turner RC, Kamundi DA, Manyama PA (2009). Red List of South African Plants 2009.

[b48] Rodrigues ASL, Gaston KJ (2002). Optimisation in reserve selection procedures – why not?. Biological Conservation.

[b49] Srivastava DS, Cadotte MW, MacDonald AAM, Marushia RG, Mirotchnick N (2012). Phylogenetic diversity and the functioning of ecosystems. Ecology Letters.

[b50] Underhill LG (1994). Optimal and suboptimal reserve selection algorithms. Biological Conservation.

[b51] Vanewright RI, Humphries CJ, Williams PH (1991). What to protect – Systematics and the agony of choice. Biological Conservation.

[b52] Watterson GA (1975). On the number of segregating sites in genetical models without recombination. Theoretical Population Biology.

[b53] Winter M, Devictor V, Schweiger O (2013). Phylogenetic diversity and nature conservation: where are we?. Trends in Ecology & Evolution.

[b54] Yang ZH, Rannala B (1997). Bayesian phylogenetic inference using DNA sequences: a Markov Chain Monte Carlo method. Molecular Biology and Evolution.

