# Supplementary material for: Split diversity in constrained conservation prioritization using integer linear programming
Source: Methods Ecol Evol. 2014 Dec 6;6(1):83–91. doi: 10.1111/2041-210X.12299 (PMC4392707; doi:10.1111/2041-210X.12299)
Supplement: Data S1 — Reserve Selection Problems Revisited. Data S2. Data description. Data S3. Additional results and analysis. Table S1. Diet composition matrix for 250 Caribbean species. Table S2. NCBI accession numbers of six genes for Caribbean case study. Table S3. Sequence data collected for Caribbean case study. Table S4. List of taxa in optimal sets SPD, SSD, S1, S2 for Caribbean case study. Fig. S1. QDS chosen by the minimum representation problem and the spatial reserve selection for the Cape of South Africa. Fig. S2. QDS determined by Marxan for the minimum representation problem. Fig. S3. QDS for W2, W3, W4. Fig. S4. QDS that host the threatened genera Polhillia, Clivia, Daubenya, and Marasmodes. Fig. S5. Food web restricted to only those taxa present in SPD or SSD. Fig. S6. Dependence of SD on subset size, k, and diet portion, d. Fig. S7. Gene trees for 12S rRNA, 16S rRNA, 18S rRNA, 28S rRNA, COI, CYTB. Fig. S8. Tree for supermatrix of six genes. Fig. S9. Super split network of six gene trees. [file mee30006-0083-sd1.pdf]

Supplementary Material

Integer Programming for Biodiversity Optimization

The supplementary material includes:

		Page
Data S1:	Reserve Selection Problems Revisited	2
Data S2:	Data description<br>*Study Areas, Sequence Data, and Phylogenetic Inference*	4
Data S3:	Additional results and analysis	6
Table S1:	Diet composition matrix for 250 Caribbean species.	*
Table S2:	NCBI accession numbers of six genes for Caribbean case study.	*
Table S3:	Sequence data collected for Caribbean case study.	11
Table S4:	List of taxa in optimal sets S_{PD}, S_{SD}, S_1, S_2 for Caribbean case study.	12
Figure S1:	QDS chosen by the minimum representation problem and the spatial reserve selection for the Cape of South Africa.	13
Figure S2:	QDS determined by Marxan for the minimum representation problem.	14
Figure S3:	QDS for W_2, W_3, W_4.	15
Figure S4:	QDS that host the threatened genera Polhillia, Clivia, Daubenya, and Marasmodes.	16
Figure S5:	Food web restricted to only those taxa present in S_{PD} or S_{SD}.	17
Figure S6:	Dependence of SD on subset size, k, and diet portion, d.	18
Figure S7:	Gene trees for 12S rRNA, 16S rRNA, 18S rRNA, 28S rRNA, COI, CYTB **	19-24
Figure S8:	Tree for supermatrix of six genes **	25
Figure S8:	Super split network of six gene trees	26

* The data is available in a separate excel file.

** Note that here the trees are drawn as rooted, but we treated them as unrooted trees in the analysis.

Supplementary Data S1: Reserve Selection Problems Revisited

We now formulate the classical *minimum representation problem* in IP parlance. Let $X = \{s_1, s_2, \dots, s_n\}$ denote the set of n taxa of interest. Further, for area $i \in \{1, \dots, m\}$ let $R_i \subset X$ denote the taxa present in area i and $c_i > 0$ are the conservation costs for that area. Here, conservation costs include, e.g. the acquisition and maintenance costs of the area. The question is then:

Problem S1 (*Minimum representation*): Given a taxon set X and m areas, find the cheapest subset of areas $\mathcal{W}_{min} \subset \{1, \dots, m\}$ such that every taxon is present in at least one area of $\mathcal{W}_{min}$.

Such a set $\mathcal{W}_{min}$ represents the most cost-effective solution in which every taxon is preserved. To apply IP, we construct the $m \times n$ presence/absence matrix $\mathcal{R}$, where $r_{ij} = 1$ if taxon s_j is present in area i, and $r_{ij} = 0$ otherwise. Moreover, we encode a subset $\mathcal{W}$ of areas by the binary vector $(x_1, x_2, \dots, x_m)$, where $x_i = 1$ if $i \in \mathcal{W}$, and $x_i = 0$ otherwise. The minimum representation problem is equivalent to determining $x_1, x_2, \dots, x_m$ such that the objective function:

$$mrp(\boldsymbol{x}) = \sum_{i=1}^{m} c_i x_i \quad (1.1)$$

is minimal, subject to

$$\sum_{i=1}^{m} r_{ij} x_i \geq 1, \quad \forall j = 1,2, \dots, n \; (1.2)$$

$$x_i \in \{0,1\}, \qquad \forall i = 1,2, \dots, m$$

The objective function (1.1) provides the total cost of the selected areas. The representation constraints (1.2) guarantee that every taxon s_j is present in at least one area.

The solution of the minimum representation problem can lead to highly fragmented areas, which causes isolated populations and reduced management efficiency (Possingham, Ball & Andelman 2000). The *spatial reserve selection* (Moilanen, Wilson & Possingham 2009) resolves this issue by additionally minimizing the cost of fencing the entire selected areas.

Problem S2 (*Spatial reserve selection*): Given a taxon set X, the presence/absence matrix $\mathcal{R}$ and the costs per unit boundary, find the cheapest subset of areas $\mathcal{W}_{min}$ such that every taxon is present in at least one area.

To quantify the boundary length we define a symmetric $m \times m$ matrix with each entry b_{ij} being the shared boundary length between area i and j. If i and j are not adjacent we set $b_{ij} = 0$. Finally, b_{ii} equals the perimeter of i. β defines the conservation cost per unit boundary length. Problem S2 was formulated as *integer quadratic programming* (Possingham, Ball & Andelman 2000):

Minimize:

$$\sum_{i=1}^{m} c_i x_i + \beta \left(\sum_{i=1}^{m} b_{ii} x_i - 2 \sum_{i=1}^{m-1} \sum_{j=i+1}^{m} b_{ij} x_i x_j \right) \quad (2.1)$$

Subject to:

$$\sum_{i=1}^{m} r_{ij} x_i \geq 1, \quad \forall j = 1,2,\dots,n$$

$$x_i \in \{0,1\}, \qquad \forall i = 1,2,\dots,m$$

Compared to problem S1 the objective function has an extra summand to include the cost β of fencing multiplied with the total boundary length. If $\beta = 0$, problem S2 reduces to problem S1. Larger β will penalize solutions consisting of isolated areas with high boundary length.

Due to the non-linear objective function, heuristic methods such as the greedy algorithm and simulated annealing were suggested (Pressey, Possingham &

Margules 1996; Possingham, Ball & Andelman 2000). However, problem S2 can be reformulated as IP (Önal & Briers 2002; Önal & Briers 2003): For each pair of adjacent areas i and j ($b_{ij} > 0$) we introduce the variable z_{ij}, where $z_{ij} = 1$ if $x_i = x_j = 1$ and $z_{ij} = 0$, otherwise (i.e., $z_{ij} \equiv x_i x_j$). In order to get the IP formulation of problem S2 we simply substitute $x_i x_j$ by z_{ij} in (2.1) and include two additional constraints for new variables

$$z_{ij} \leq x_i \text{ and } z_{ij} \leq x_j, \quad \forall i, j = 1,2, \dots, m$$

which ensure that $z_{ij} \equiv x_i x_j$. Note that the number of introduced variables z_{ij} is typically much smaller than $\binom{m}{2}$ as only pairs of spatially adjacent areas are relevant.

Supplementary Data S2: Data description

Study Areas, Sequence Data, and Phylogenetic Inference

Case Study I: The Cape of South Africa

The first data set comprises 735 angiosperm genera from the Cape of South Africa (Forest *et al.* 2007). The Cape was partitioned into 201 quarter-degree squares (QDS), each with occurrence data for all genera. The phylogenetic reconstruction was based on plastid DNA sequences from the large subunit of the ribulose-bisphosphate carboxylase gene (*rbcL)* for the 735 genera (Forest *et al.* 2007). From the *rbcL* sequence alignment we reconstructed a maximum-likelihood (ML) tree (Minh *et al.* 2005) under the GTR+Γ model (Tavare 1986; Yang 1994). We then performed a parametric bootstrap (Felsenstein 2004) with 100 replicates to infer 100 ML bootstrap trees. The bootstrap trees obtained were joined into a union split system (Minh, Klaere & von Haeseler 2009) by extracting all splits (edges) from the trees and assigning split weights as the

average length of the corresponding edge across the trees having the split. The resulting union split system served as input to compute split diversity (see below).

Case Study II: Caribbean Coral Reef Community

The second case study deals with a well-resolved Caribbean marine food web (Opitz 1996). This data set consists of 250 taxonomic units including 208 fish species, a category "unidentified fishes", 35 non-fish taxa, two aggregated groups of consumers (zooplankton and microfauna) and four categories of primary producer: organic matter (including particulate organic matter, dissolved organic matter and detritus), benthic autotrophs, symbiotic algae and phytoplankton. We removed unidentified fishes from the food web, as well as *Synodus synodus*, which only feeds on this group.

The food web was represented as a weighted directed graph, where the nodes in the graph correspond to the taxa and the arrows point from predators to their preys. Arrow weights are defined as the fraction a prey contributes to the diet of the predator. The food web contained 20 cycles including 11 cases of cannibalism and 9 cycles of two taxa (mutual predation). Therefore, we excluded 20 arrows to obtain a directed acyclic graph. The reduced food web has 248 nodes and 3281 arrows. Finally, we rescaled the weights such that for each predator the diet sums up to 100%.

For each of the taxa we then retrieved if available four ribosomal RNA genes (12S rRNA, 16S rRNA, 18S rRNA, 28S rRNA), cytochrome c oxidase I (COI) and cytochrome b (CYTB) from the NCBI database (Geer *et al.* 2010). If the taxa corresponded to a genus or family, we chose one representative species having most sequences from this genus or family. When none of the genes were

available for a given species, we substituted this species with its closest relative from the same genus (or family) (the complete list of species and the NCBI accession numbers are available in Supplementary Table S2). For each rRNA gene we aligned sequences using MAFFT L-INS-i v6.935b (Katoh & Toh 2008). For COI and CYTB we used TranslatorX (Abascal, Zardoya & Telford 2010) and MAFFT to align the translated amino acid sequences and then back translate into cDNA alignments.

Finally, we reconstructed a maximum-likelihood (ML) tree for each gene using IQ-TREE (Minh, Nguyen & von Haeseler 2013), where the substitution models were selected by the Bayesian information criterion (Schwarz 1978). From the gene trees we reconstructed a split network using SplitsTree4 (Huson *et al.* 2004; Huson & Bryant 2006), and used the split network to compute the SD. Moreover, we concatenated the six gene alignments into a super alignment and inferred the ML tree from this super alignment. This *species* tree is used to compute the optimal set of species based on PD and compare with the results obtained using SD.

Supplementary Data S3: Additional results and analysis

Case Study I: The Cape of South Africa

Selecting Representative QDS

We start to demonstrate the utility of IP with the classical minimum representation problem (Problem 1; Material and Methods). In short, a set of QDS is sought that will preserve all genera while minimizing the costs, where the cost of a QDS is simply equal to its area size. For the Cape, the best solution comprises 28 QDS (marked in blue and hatched; Supplementary Fig. S1) for a total of about 13,000 km^2, one QDS smaller than the set previously suggested by

a greedy algorithm (Forest *et al.* 2007). The selected QDS cover areas including the Cape Peninsula near Cape Town, a region comprising afrotemperate forest vegetation along the South-East coast, the surrounding area of Port Elizabeth and the Bokkeveld plateau near Nieuwoudtville. Despite the presence of one of the largest cities in South Africa on the peninsula (Cape Town), a large part of the land surface and unique biodiversity covered by these QDS is part of the Table Mountain National Park, which also includes the renowned Cape of Good Hope, and which consists in large part of sandstone fynbos vegetation (Mucina & Rutherford 2006). An area further east along the coast includes several QDS containing a large part of the total afrotemperate forest vegetation of the Cape, which present a much different species composition than the surrounding fynbos vegetation. The most eastern QDS selected, covering an area surrounding the city of Port Elizabeth, is notable for the presence of Albany thicket biome vegetation, a vegetation type also found in other parts of the Cape, but more prominent in the eastern part of the region. Finally, QDS selected in the Northwestern part of the region overlap with the surroundings of the Bokkeveld plateau, an area particularly renowned for its rich diversity of geophyte species (i.e. species with underground storage structures).

The 28 representative QDS are, however, highly fragmented, an undesirable situation for conservation planning (Possingham, Ball & Andelman 2000). High fragmentation also means long boundaries, making the whole area more vulnerable to outside factors such as human activities or weed and pest invasions. Alternatively, we minimize the total land mass and the boundary length simultaneously, the so-called spatial reserve selection (Problem 2; Material and Methods). Under this scenario, the optimal solution of 30 QDS

(marked in magenta and hatched; Supplementary Fig. S1) will host all genera. While the land area is increased by almost 2,000 km^2, the total border length is reduced by nearly 250 km due to the selection of neighbouring QDS like the nature reserves to the east of Cape Town (e.g. Kogelberg) and the afrotemperate forests along the southern coast line.

Case Study II: Caribbean Coral Reef Community

We first compared taxon selection results for PD and SD measures without taking into account the food web. S_{PD} denotes a set of k taxa having maximal PD and S_{SD} a set of k taxa having maximal SD. We require that the six aggregate groups are always present in S_{PD} and S_{SD}, both because they form the base of the food web and because they represent taxonomically diverse collections of organisms rather than species or even higher taxonomic levels. We obtained S_{PD} by the greedy algorithm (Minh, Klaere & von Haeseler 2006), and S_{SD} using IP (Minh, Klaere & von Haeseler 2010). For $k = 24$ (10% of the taxa), S_{PD} (red and blue nodes; food web restricted to S_{PD} contains red and blue arrows; Supplementary Fig. S5) has a relative PD of 36.12% compared to the total branch lengths in the phylogenetic tree. That is, 10% of the species in the Caribbean food web conserves 36.12% of its evolutionary history.

In the split system the optimal subset S_{SD} (green and blue nodes; food web restricted to S_{SD} contains green and blue arrows; Supplementary Fig. S5) with 24 taxa conserves 57.89% of the total SD (i.e. the sum of the all split weights). S_{PD} and S_{SD} only share 11 taxa (blue nodes; Supplementary Fig. S5). The other 13 species in each set are different and belong to different genera (red and green nodes for species exclusively in S_{PD} and S_{SD}, respectively; Supplementary Fig. S5).

We note that *Synodus foetens* (in S_{PD}) and *Antennarius striatus* (in both sets S_{PD} and S_{SD}) have no outgoing arrows in Supplementary Fig. S5. This renders S_{PD} and S_{SD} inviable, and illustrates the need to include information about ecological dependencies in conservation decisions.

We also exhaustively computed all optimal SD sets $S_{max}(k, d)$ for $k \in \{10\%, 20\%, \dots, 90\%\}$ and $d \in \{0\%, 1\%, \dots, 90\%\}$. Supplementary Fig. S6 shows the optimal SD of all these sets. For a fixed k the changes in the SD of $S_{max}(k, d)$ with varying d are moderate as indicated by almost horizontal lines in Supplementary Fig. S6. The largest difference is 4.63% for $k = 10\%$ and d increasing from 0% to 90%. That means, even stringent viability constraints with high d still provide us almost equally optimal subsets.

Computational time

The computational time to solve the problems with PDA software was less than 8 seconds on a 2.66 GHz computer. 98% of the runs for different parameters of problem 4 consumed less than 1 second with a maximum of 3 seconds. Simulations with varying complexities of split networks and food webs yielded average run times of 2 seconds, with a maximum of 8 seconds.

Analysis with Marxan software

Finally, we solved problems S1 and S2 with the software Marxan 2.1.1 (Ball, Possingham & Watts 2009). We used Marxan to solve problems S1 and S2 for 50 instances each (following the recommended setting per run: 1 million iterations per run, and an adaptive cooling scheme). The solution of every run was improved by a greedy and iterative improvement heuristics (Pressey, Possingham & Day 1997). For problem S1 only 7 Marxan runs found the optimal solutions whereas the remaining 43 runs returned sub-optimal solutions. The 7

runs returned 3 distinct optimal solutions; one is identical to our set of QDS and the remaining two only differ by one QDS (Supplementary Fig. S2). For problem S2 Marxan found only optimal solution in 3 runs (i.e., sub-optimal solutions in 47 runs).

Note that it was not possible to perform the analysis with Marxan for problems 1-4 because it works with neither PD nor SD.

Supplementary Table S3. Sequence data collected for the second case study.

Genes	No. Sequences	No. Sites	Substitution model
12S rRNA	115	1328	GTR+G
16S rRNA	171	2680	GTR+I+G
18S rRNA	24	2887	TN+G
28S rRNA	51	4791	GTR+G
Cytochrome c oxidase I (COI)	218	1641	GTR+I+G
Cytochrome b (CYTB)	130	1173	TVM+I+G
Super matrix	242	14500	GTR+G

Supplementary Table S4. List of taxa in optimal sets S_{PD}, S_{SD}, S_1, S_2 to preserve 24 (10%) taxa. Numbers in parentheses depict the diversity percentages of the set compared to the total diversity. Table entries show the sum of diet proportions for each taxon over other taxa in the same set. Minus signs indicate the absence of the taxa from the corresponding set.

Taxon name	S_{PD} (36.12%)	S_{SD} (57.89%)	S_1 (57.67%)	S_2 (56.36%)
Organic matter	0	0	0	0
Benthic autotrophs	0	0	0	0
Symbiotic algae	0	0	0	0
Phytoplankton	0	0	0	0
Zooplankton	1	1	1	1
Microfauna	1	1	1	1
Ophioblennius atlanticus	-	1	1	1
Acanthopleura granulata	-	0.84	0.84	-
Quadrimaera pacifica	1	1	1	1
Haemulon plumierii	-	0.5	0.5	0.5
Holothuria floridana	1	1	1	1
Achelia sawayai	-	0.2	0.2	0.35
Sphyraena barracuda	-	0.06	0.06	-
Galeocerdo cuvier	-	0.11	0.11	-
Stegastes planifrons	-	0.58	0.58	0.58
Elacatinus evelynae	-	-	1	-
Holacanthus ciliaris	-	0.02	0.02	0.98
Panulirus argus	-	0.3	0.3	0.7
Stenopus hispidus	-	0.66	0.66	0.67
Ophiocoma echinata	1	1	1	1
Octopus vulgaris	-	0.07	0.07	-
Idotea baltica	1	1	1	1
Pagurus longicarpus	-	0.2	0.2	1
Callinectes sapidus	-	0.81	0.81	0.86
Carcharhinus leucas	-	-	-	0.3
Sphyraena picudilla	-	-	-	0.69
Pinctada radiata	-	-	-	1
Iotrochota birotulata	-	-	-	1
Sepioteuthis sepioidea	0.75	-	-	1
Reteporella beaniana	1	-	-	-
Synodus foetens	0	-	-	-
Fregata magnificens	1	-	-	-
Cittarium pica	0.86	-	-	-
Synodus intermedius	0.05	-	-	-
Loimia medusa	0.93	-	-	-
Millepora sp. AMN-2008	1	-	-	-
Cephalodiscus gracilis	1	-	-	-
Megabalanus californicus	1	-	-	-
Oreaster reticulatus	0.83	-	-	-
Graptacme eborea	1	-	-	-
Gorgonia flabellum	1	-	-	-
Antennarius striatus	0	0	-	-

Supplementary Figure S1. Representative QDS required to preserve all sampled plant genera of the Cape of South Africa for the minimum representation problem (hatched and blue squares) and the spatial reserve selection (hatched and magenta squares), respectively.

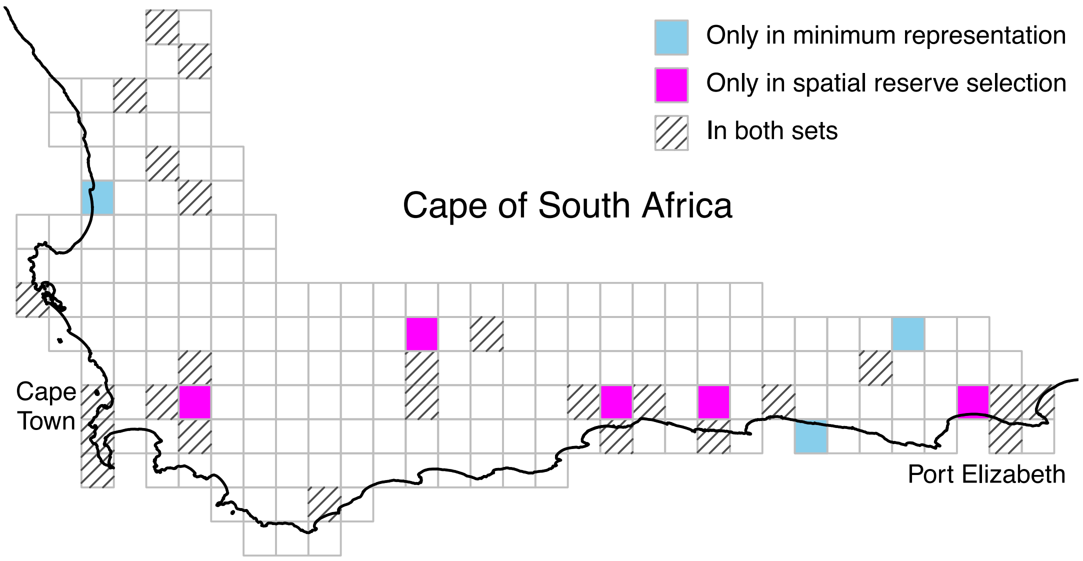

Supplementary Figure S2. QDS determined by Marxan for the minimum representation problem. Seven of 50 Marxan runs converged to three equally optimal solutions, each comprises 27 grey QDS and one of the three blue QDS.

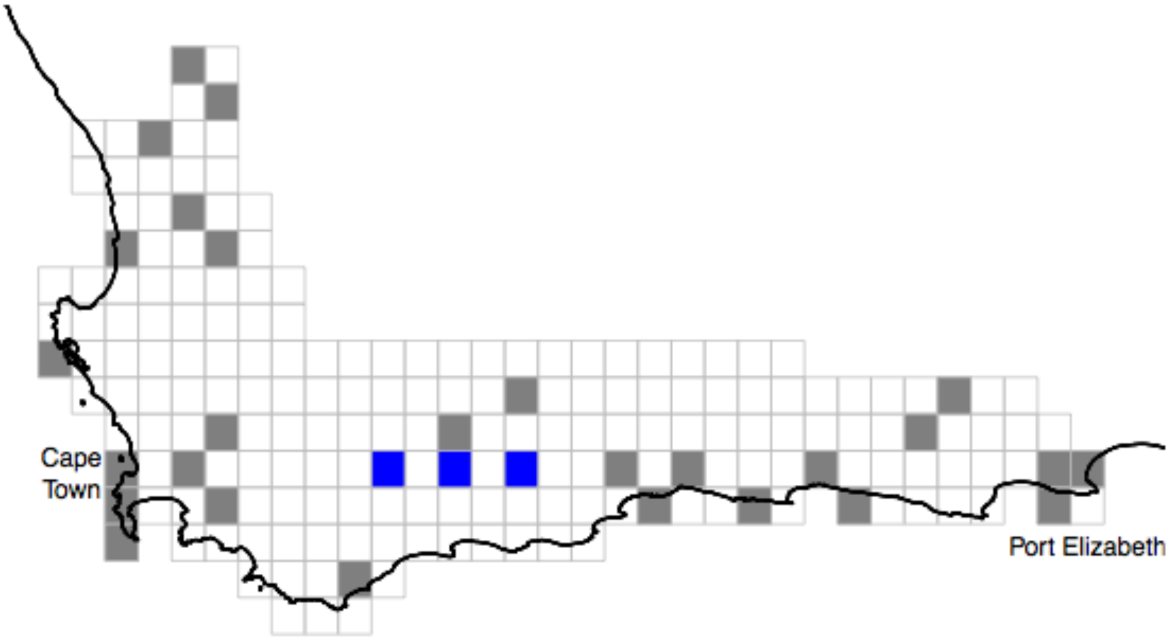

Supplementary Figure S3. QDS for W_2, W_3, W_4.

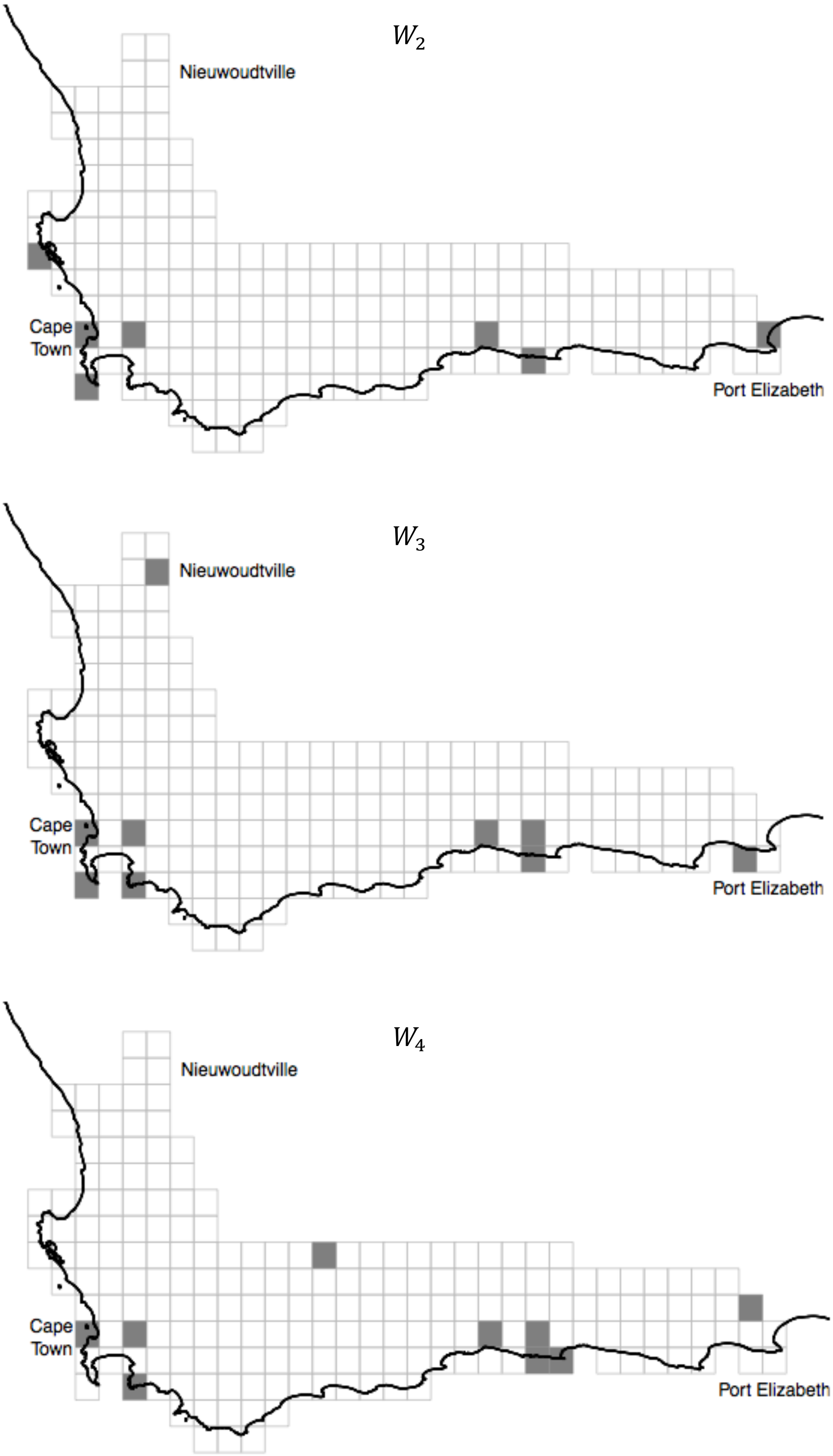

Supplementary Figure S4. QDS that host the threatened genera Polhillia, Clivia, Daubenya, and Marasmodes encoded with P, C, D, and M, respectively.

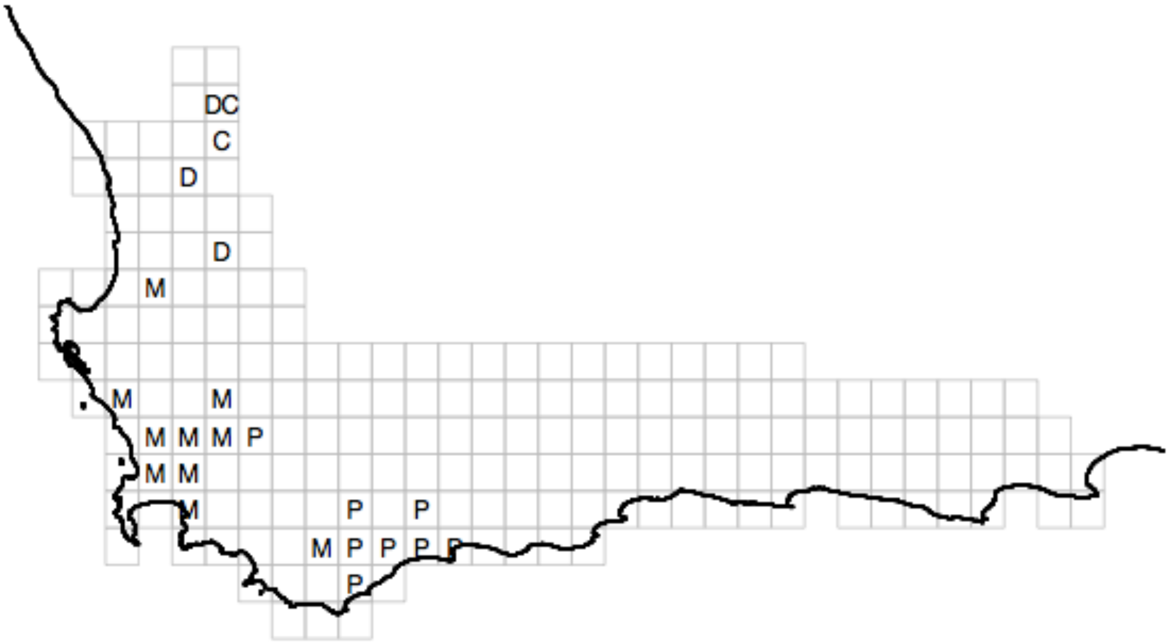

Supplementary Figure S5. Food web restricted to only those taxa present in S_{PD} or S_{SD} (see Supplementary Data S3). Red, green, and blue nodes depict the taxa present exclusively in S_{PD}, exclusively in S_{SD}, and in both sets, respectively. Light blue nodes correspond to aggregated groups. Arrows connect from predators to their preys with thickness reflecting the prey proportion in the predator diet. Arrows pointing to or from green and red nodes are colored green and red respectively. Arrows between blue nodes are colored blue. Note that the arrows between green and red nodes are ignored.

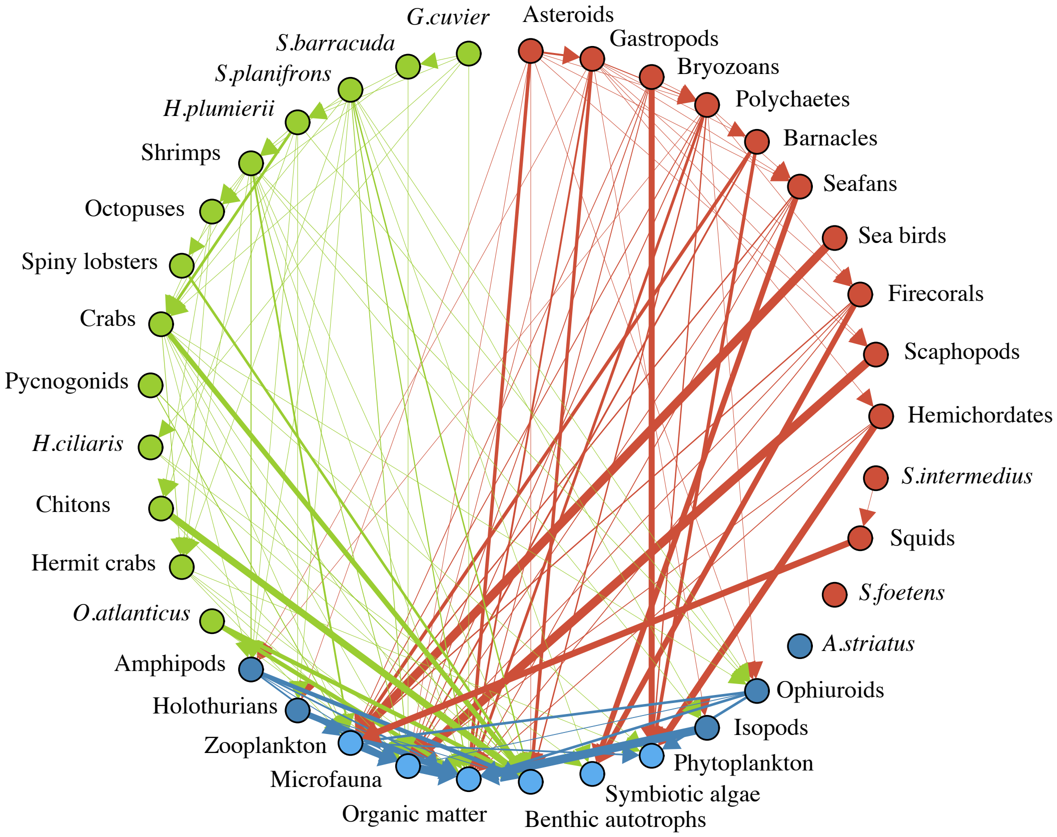

Supplementary Figure S6. Dependence of SD on subset size, k, and diet portion, d. SD of the optimal set $S_{max}(k, d)$ for varying k and d.

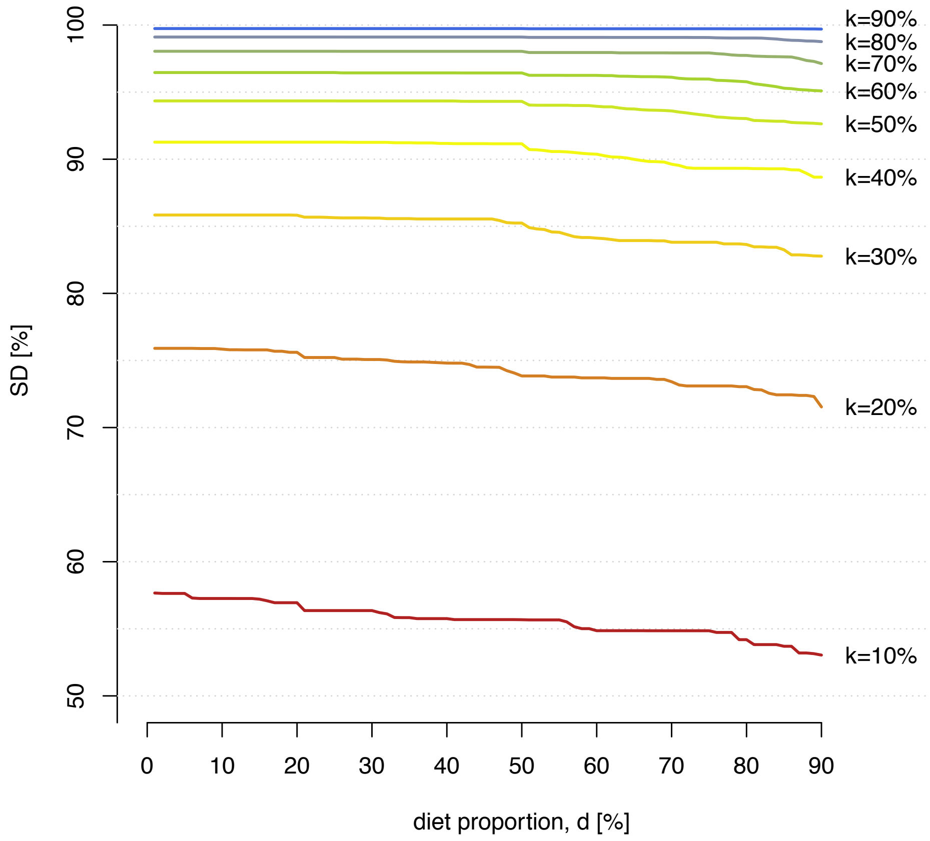

Supplementary Figure S7. Gene trees
A. Gene tree for 12S rRNA

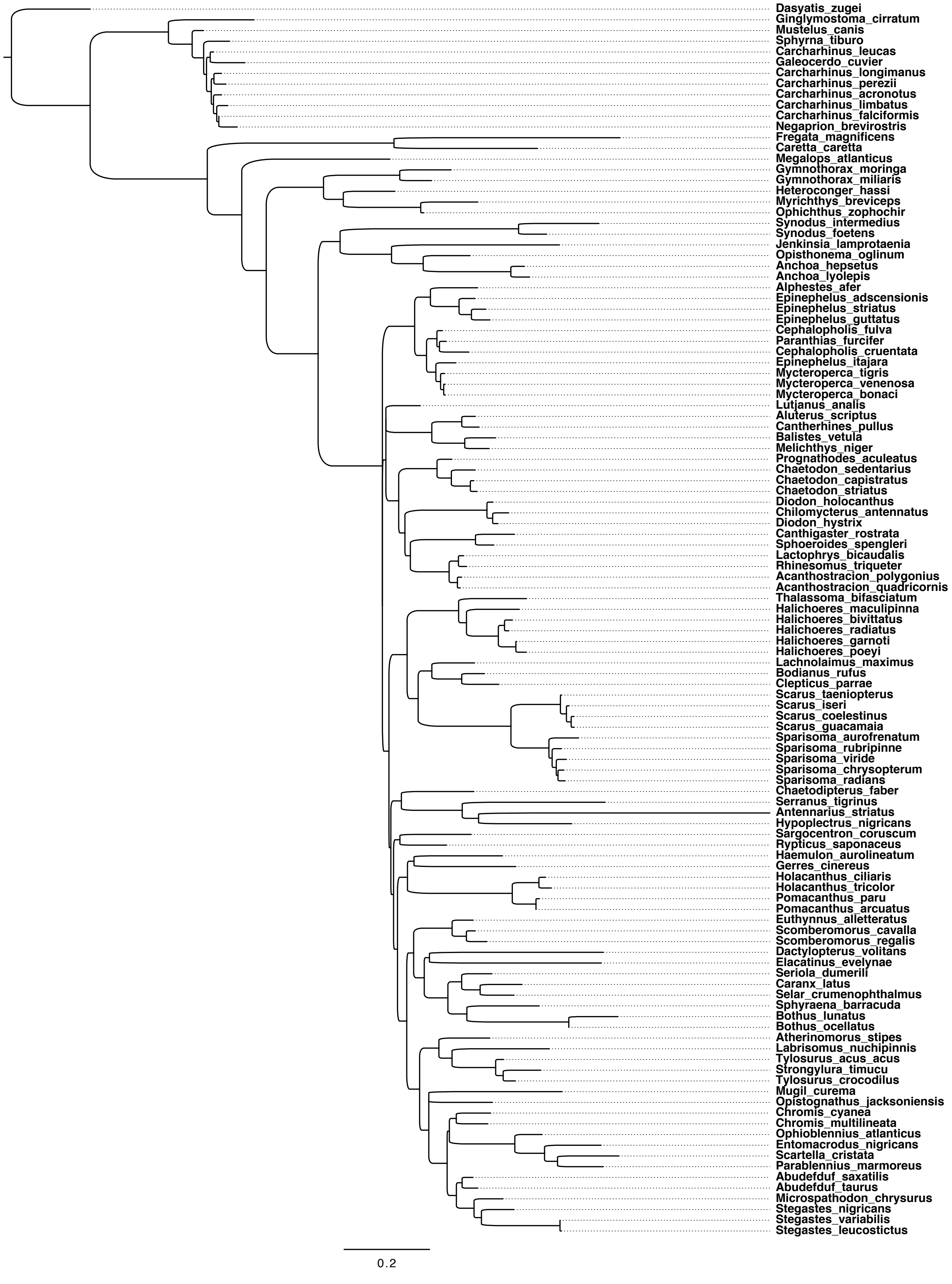

B. Gene tree for 16S rRNA

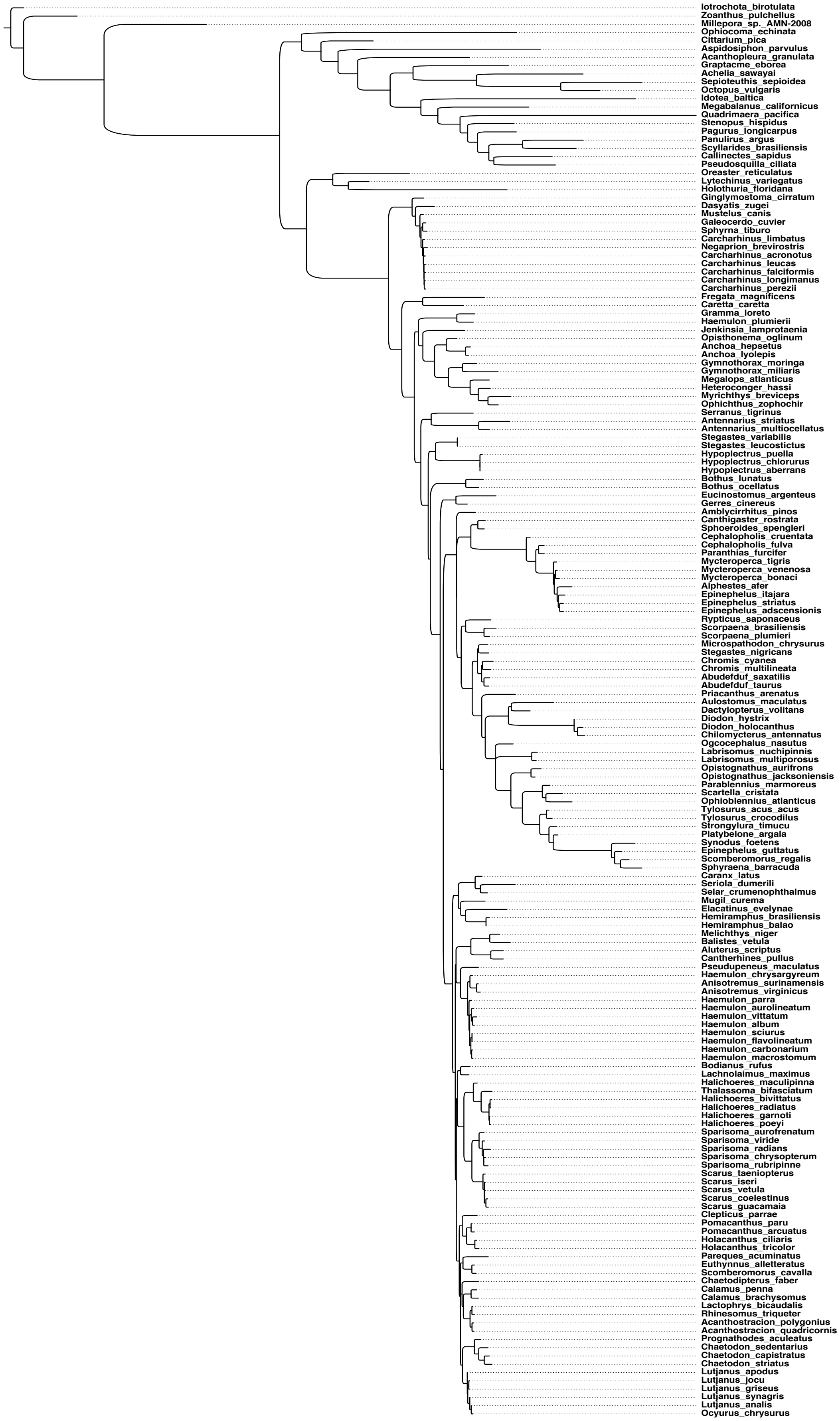

C. Gene tree for 18S rRNA

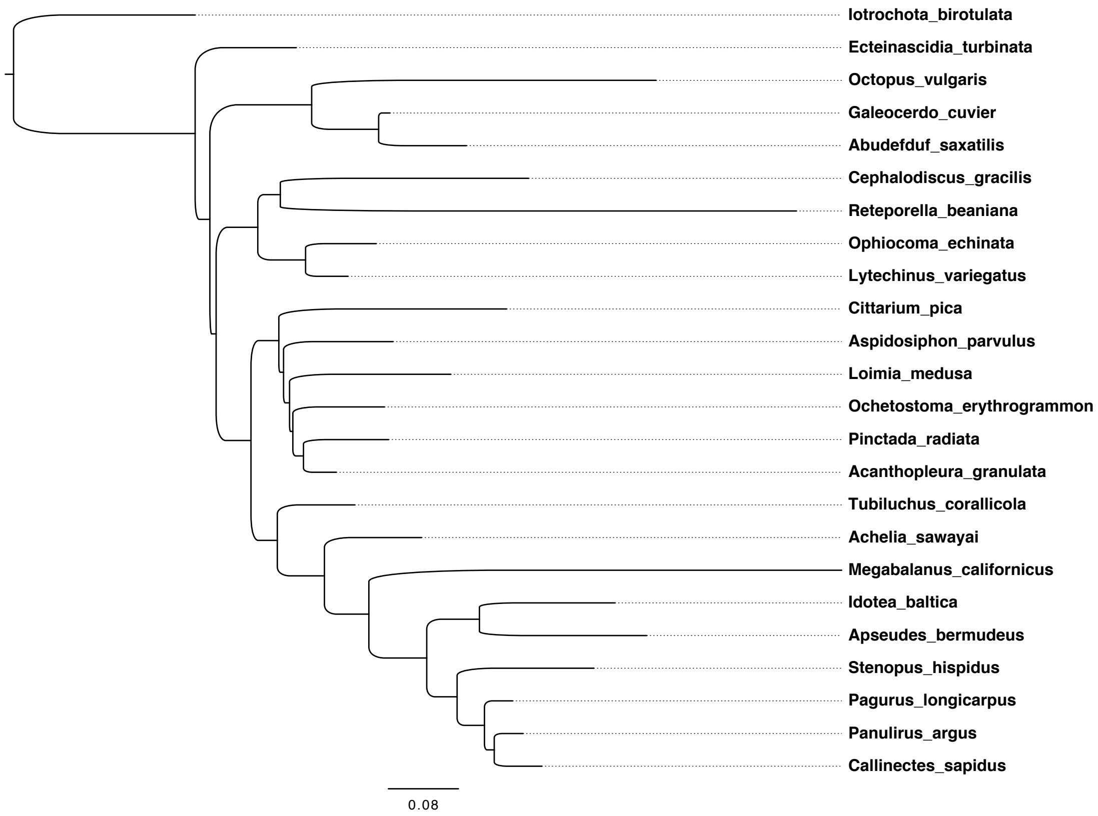

D. Gene tree for 28S rRNA

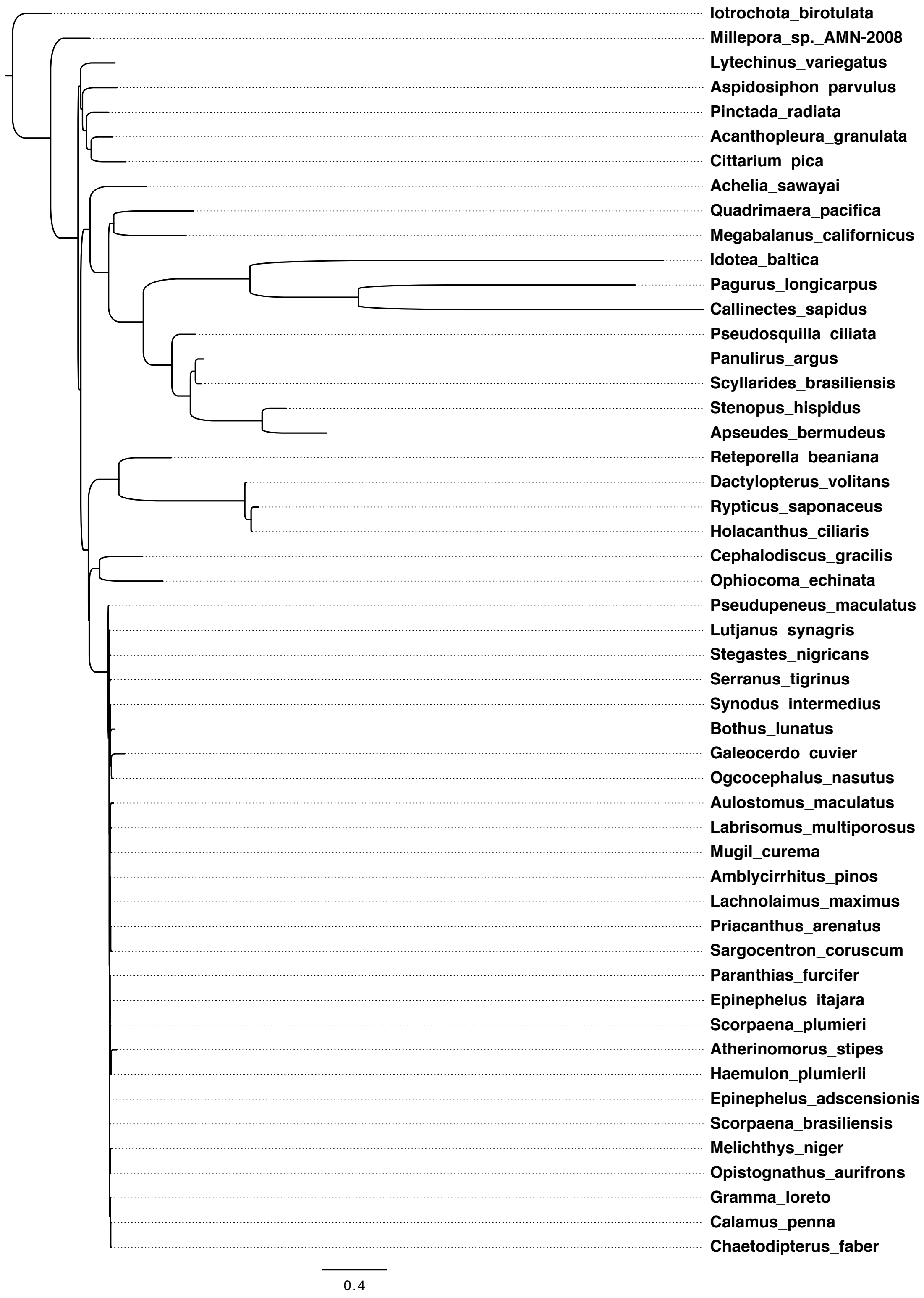

E. Gene tree for COI

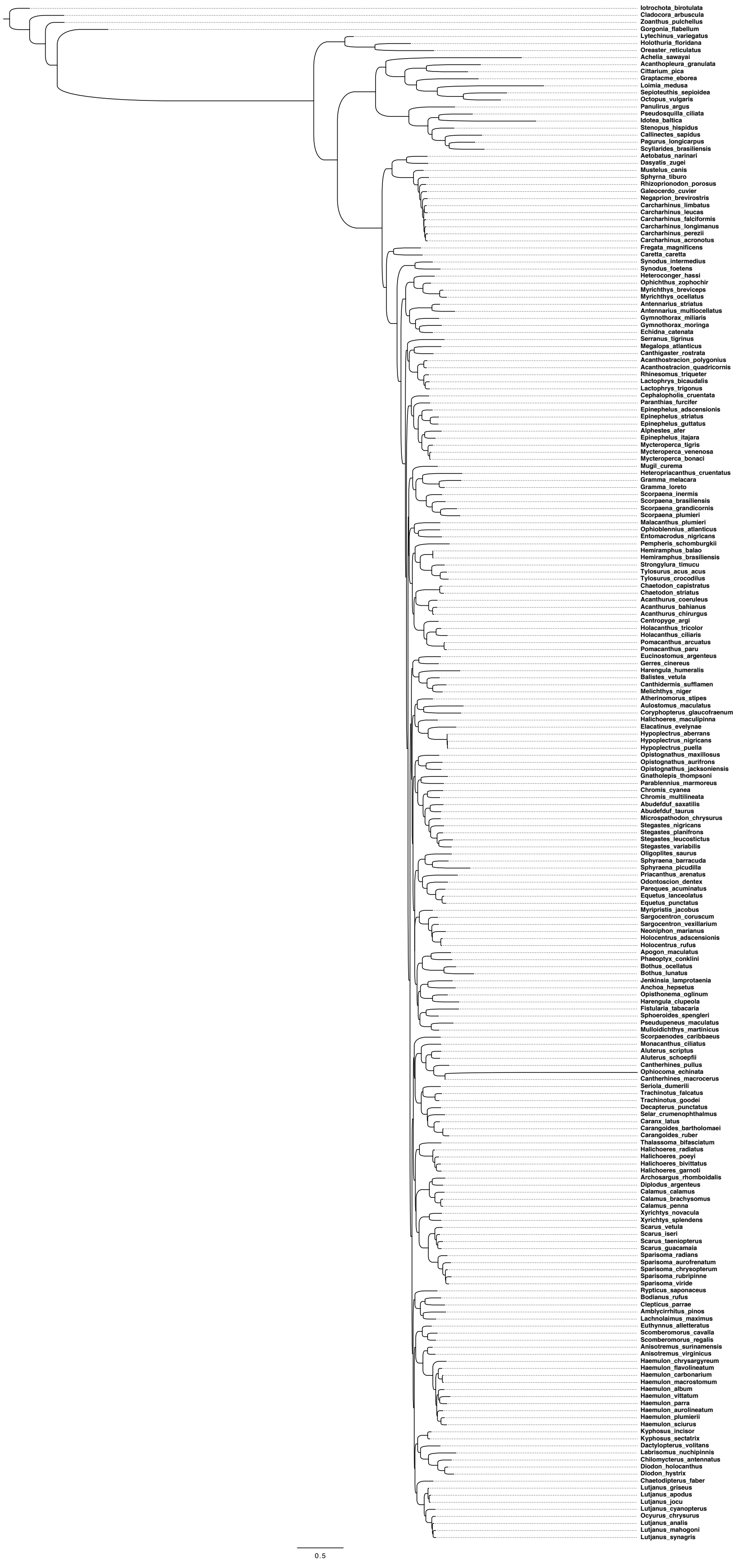

F. Gene tree for CYTB

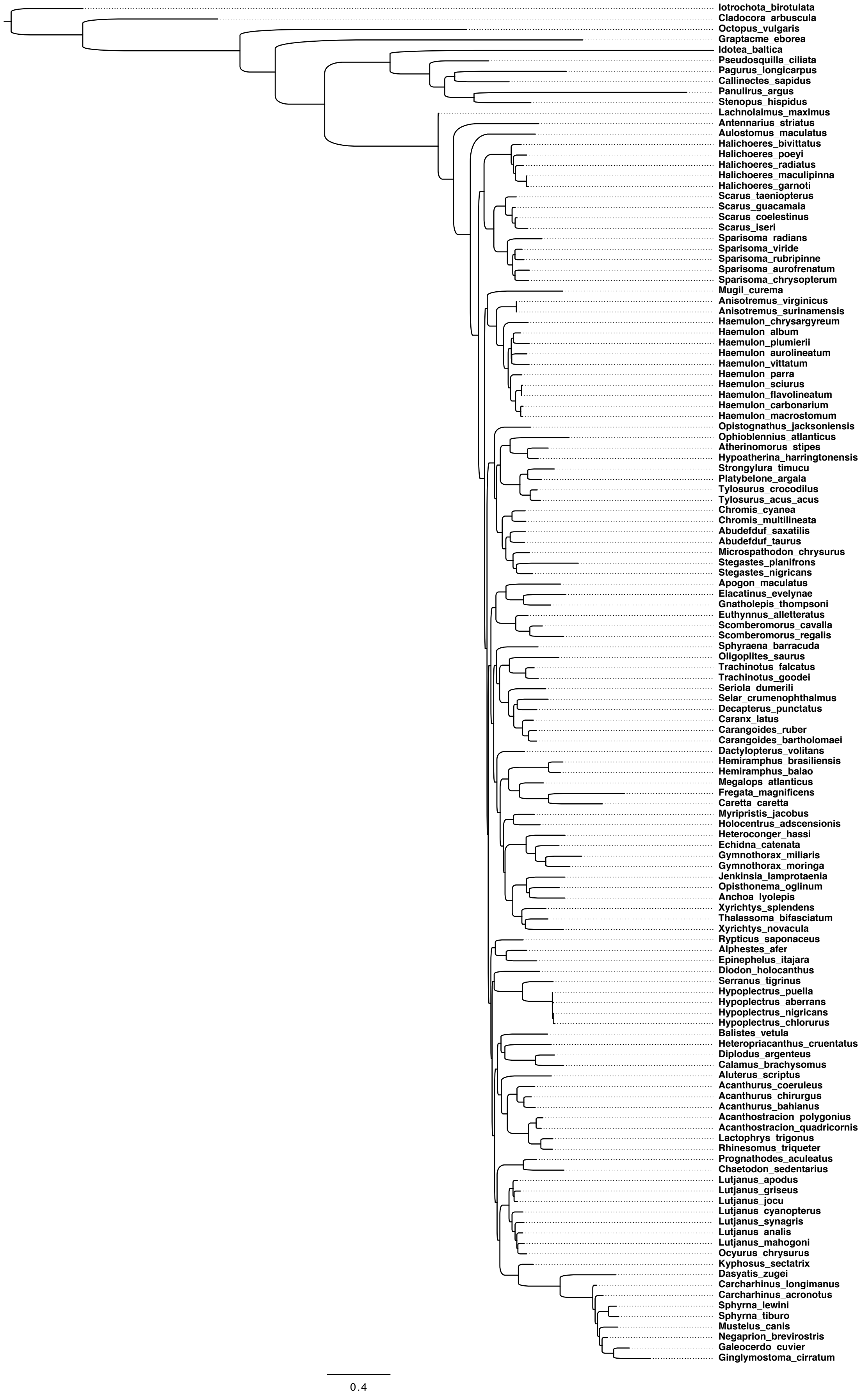

Supplementary Figure S8. Tree for supermatrix of six genes.

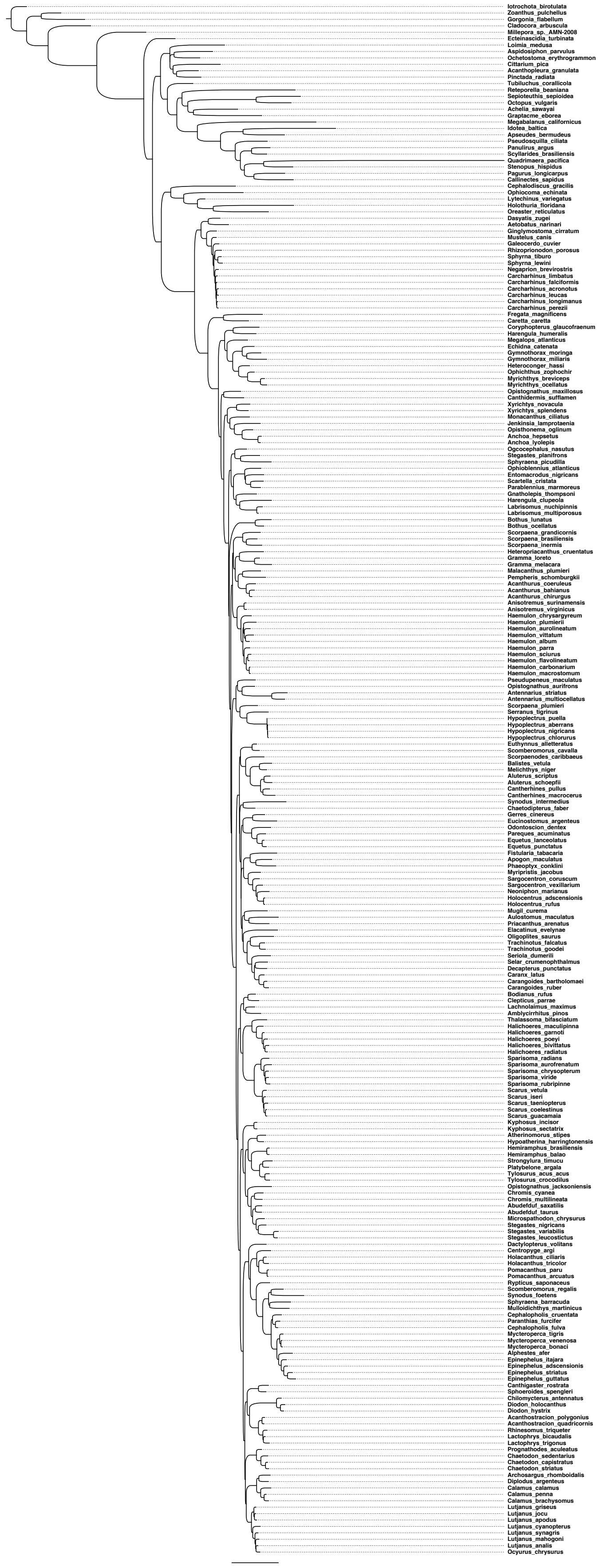

Supplementary Figure S9. Super split network of six gene trees.

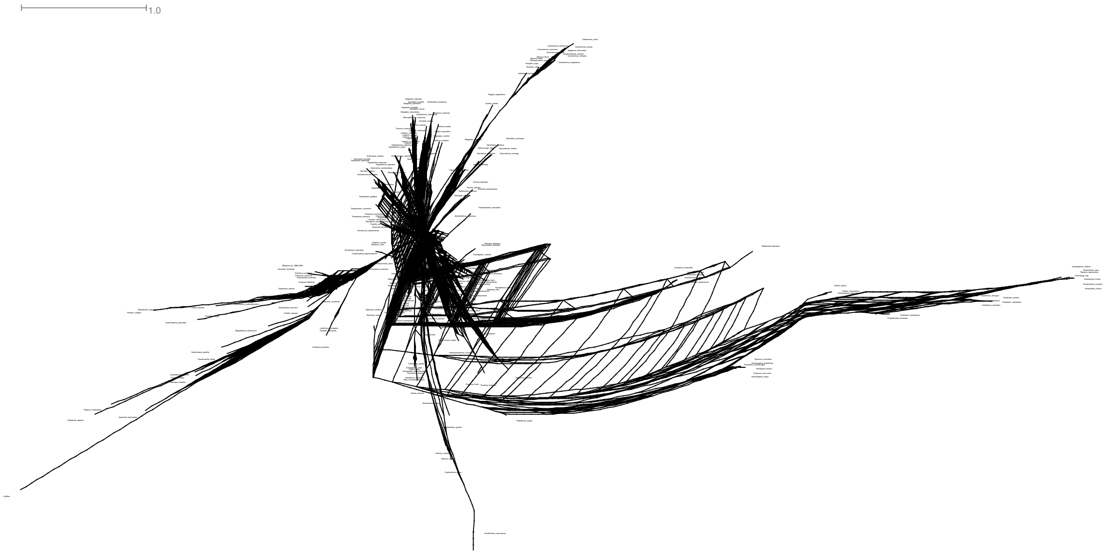

References

Abascal, F., Zardoya, R. & Telford, M.J. (2010) TranslatorX: multiple alignment of nucleotide sequences guided by amino acid translations. *Nucleic Acids Research,* **38,** W7-W13.

Felsenstein, J. (2004) *Inferring Phylogenies*. Sinauer Associates, Sunderland, Massachusetts.

Forest, F., Grenyer, R., Rouget, M., Davies, T.J., Cowling, R.M., Faith, D.P., Balmford, A., Manning, J.C., Proches, S., van der Bank, M., Reeves, G., Hedderson, T.A.J. & Savolainen, V. (2007) Preserving the evolutionary potential of floras in biodiversity hotspots. *Nature,* **445,** 757-760.

Geer, L.Y., Marchler-Bauer, A., Geer, R.C., Han, L.Y., He, J., He, S.Q., Liu, C.L., Shi, W.Y. & Bryant, S.H. (2010) The NCBI BioSystems database. *Nucleic Acids Research,* **38,** D492-D496.

Huson, D.H. & Bryant, D. (2006) Application of phylogenetic networks in evolutionary studies. *Molecular Biology and Evolution,* **23,** 254-267.

Huson, D.H., Dezulian, T., Klopper, T. & Steel, M.A. (2004) Phylogenetic super-networks from partial trees. *Ieee-Acm Transactions on Computational Biology and Bioinformatics,* **1,** 151-158.

Katoh, K. & Toh, H. (2008) Recent developments in the MAFFT multiple sequence alignment program. *Briefings in Bioinformatics,* **9,** 286-298.

Minh, B.Q., Klaere, S. & von Haeseler, A. (2006) Phylogenetic diversity within seconds. *Systematic Biology,* **55,** 769-773.

Minh, B.Q., Klaere, S. & von Haeseler, A. (2009) Taxon selection under split diversity. *Systematic Biology,* **58,** 586-594.

Minh, B.Q., Klaere, S. & von Haeseler, A. (2010) SDA*: A simple and unifying solution to recent bioinformatic challenges for conservation genetics. *The second international conference on knowledge and systems engineering* (eds S.B. Pham, T.H. Hoang, B. McKay & K. Hirota), pp. 33-37. IEEE Computer Society, Hanoi, Vietnam.

Minh, B.Q., Nguyen, M.A. & von Haeseler, A. (2013) Ultrafast approximation for phylogenetic bootstrap. *Molecular Biology and Evolution,* **30,** 1188-1195.

Minh, B.Q., Vinh le, S., von Haeseler, A. & Schmidt, H.A. (2005) pIQPNNI: parallel reconstruction of large maximum likelihood phylogenies. *Bioinformatics,* **21,** 3794-3796.

Mucina, L. & Rutherford, M.C. (2006) The Vegetation of South Africa, Lesotho and Swaziland *Strelitzia 19*, pp. 816. South African National Biodiversity Institute, Pretoria.

Nguyen, L.T., Chernomor, O., Schmidt, H., Hoang, D.T., Flouri, T., Stamatakis, A., von Haeseler, A. & Minh, B.Q. (2014) IQ-TREE: Integrative and high performance phylogenetic software in light of phylogenomics. In prep.

Opitz, S. (1996) *Trophic interactions in Caribbean coral reefs*. ICLARM Technical Reports, Makati City, Philippines.

Possingham, H.P., Ball, I.R. & Andelman, S. (2000) Mathematical methods for identifying representative reserve networks. *Quantitative Methods for Conservation Biology* (eds S. Ferson & M. Burgman), pp. 291-305. Springer, New York.

Schwarz, G. (1978) Estimating Dimension of a Model. *Annals of Statistics,* **6,** 461-464.

Tavare, S. (1986) Some probabilistic and statistical problems in the analysis of DNA sequences. *Lecture Notes on Mathematical Modelling in the Life Sciences,* **17,** 57-86.

Yang, Z.H. (1994) Maximum likelihood phylogenetic estimation from DNA sequences with variable rates over sites - Approximate methods. *Journal of Molecular Evolution,* **39,** 306-314.
